# Supplementary material for: Impact of labor analgesia on mode of delivery and neonatal outcomes in Japan: A retrospective cohort study
Source: PLoS One. 2023 Apr 12;18(4):e0284368. doi: 10.1371/journal.pone.0284368 (PMC10096281; doi:10.1371/journal.pone.0284368)

PLOS ONE

Impact of labor analgesia on mode of delivery and neonatal outcomes in Japan

--Manuscript Draft--

**Manuscript Number:**	PONE-D-22-18968
**Article Type:**	Research Article
**Full Title:**	Impact of labor analgesia on mode of delivery and neonatal outcomes in Japan
**Short Title:**	Impact of labor analgesia on delivery and neonates
**Corresponding Author:**	Shu Eguchi<br>Saint Luke's International Hospital: Sei Roka Kokusai Byoin<br>Tokyo, JAPAN
**Keywords:**	labor analgesia; cesarean section; instrumental vaginal delivery; neonate
**Abstract:**	Labor analgesia (LA) is associated with the potential hazard of high-risk delivery, such as cesarean section (CS) and instrumental vaginal delivery (IVD), and adverse neonatal outcomes. Our objective was to examine the impact of LA on mode of delivery and neonatal outcomes and to identify risk factors in pregnant women who should avoid LA. This retrospective cohort study containing 5,184 pregnant women analyzed the association between LA and both mode of delivery and neonatal outcomes. LA increased the risk of IVD (Adjusted Odds Ratio [AOR] 3.25) but decreased that of CS (AOR 0.52). Four factors (advanced maternal age [AOR 1.72], lower maternal height, larger estimated fetal weight, and primipara [AOR 6.76]) increased the risk of IVD. We should carefully consider the indication of LA for cases with these four factors since LA can increase the risk of IVD and adverse neonatal outcomes.
**Order of Authors:**	Shu Eguchi
	Yuko Nagaoki
	Sachiko Ohde
	Michio Hirata
**Additional Information:**	
**Question**	**Response**
**Financial Disclosure**<br><br>Enter a financial disclosure statement that describes the sources of funding for the work included in this submission. Review the submission guidelines for detailed requirements. View published research articles from *PLOS ONE* for specific examples.<br><br>This statement is required for submission and **will appear in the published article** if the submission is accepted. Please make sure it is accurate.	The funders had no role in study design, data collection and analysis, decision to publish, or preparation of the manuscript.

Powered by Editorial Manager® and ProduXion Manager® from Aries Systems Corporation

**Unfunded studies**<br>Enter: *The author(s) received no specific funding for this work.*<br><br>**Funded studies**<br>Enter a statement with the following details:<br>• Initials of the authors who received each award<br>• Grant numbers awarded to each author<br>• The full name of each funder<br>• URL of each funder website<br>• Did the sponsors or funders play any role in the study design, data collection and analysis, decision to publish, or preparation of the manuscript?<br>• **NO** - Include this sentence at the end of your statement: *The funders had no role in study design, data collection and analysis, decision to publish, or preparation of the manuscript.*<br>• **YES** - Specify the role(s) played.<br><br>* typeset	
**Competing Interests**<br><br>Use the instructions below to enter a competing interest statement for this submission. On behalf of all authors, disclose any competing interests that could be perceived to bias this work—acknowledging all financial support and any other relevant financial or non-financial competing interests.<br><br>This statement is required for submission and **will appear in the published article** if the submission is accepted. Please make sure it is accurate and that any funding sources listed in your Funding Information later in the submission form are also declared in your Financial Disclosure statement.<br><br>View published research articles from *PLOS ONE* for specific examples.	The authors have declared that no competing interests exist.

**NO authors have competing interests**<br><br>Enter: *The authors have declared that no competing interests exist.*<br><br>**Authors with competing interests**<br><br>Enter competing interest details beginning with this statement:<br><br>*I have read the journal's policy and the authors of this manuscript have the following competing interests: [insert competing interests here]*<br><br>* typeset	
**Ethics Statement**<br><br>Enter an ethics statement for this submission. This statement is required if the study involved:<br><br>• Human participants<br>• Human specimens or tissue<br>• Vertebrate animals or cephalopods<br>• Vertebrate embryos or tissues<br>• Field research<br><br>Write "N/A" if the submission does not require an ethics statement.<br><br>General guidance is provided below. Consult the submission guidelines for detailed instructions. **Make sure that all information entered here is included in the Methods section of the manuscript.**	This study was approved by the institutional review board of our institute (Approval code: 21-R113) and was conducted according to the principles of the Declaration of Helsinki.<br><br>Since this study was an observational retrospective study, written informed consent was waived and we proceeded using an opt-out approach.

**Format for specific study types**<br><br>**Human Subject Research (involving human participants and/or tissue)**<br>• Give the name of the institutional review board or ethics committee that approved the study<br>• Include the approval number and/or a statement indicating approval of this research<br>• Indicate the form of consent obtained (written/oral) or the reason that consent was not obtained (e.g. the data were analyzed anonymously)<br><br>**Animal Research (involving vertebrate animals, embryos or tissues)**<br>• Provide the name of the Institutional Animal Care and Use Committee (IACUC) or other relevant ethics board that reviewed the study protocol, and indicate whether they approved this research or granted a formal waiver of ethical approval<br>• Include an approval number if one was obtained<br>• If the study involved *non-human primates*, add *additional details* about animal welfare and steps taken to ameliorate suffering<br>• If anesthesia, euthanasia, or any kind of animal sacrifice is part of the study, include briefly which substances and/or methods were applied<br><br>**Field Research**<br><br>Include the following details if this study involves the collection of plant, animal, or other materials from a natural setting:<br>• Field permit number<br>• Name of the institution or relevant body that granted permission	
**Data Availability**<br><br>Authors are required to make all data underlying the findings described fully available, without restriction, and from the time of publication. PLOS allows rare exceptions to address legal and ethical concerns. See the PLOS Data Policy and FAQ for detailed information.	No - some restrictions will apply

Powered by Editorial Manager® and ProduXion Manager® from Aries Systems Corporation

A Data Availability Statement describing where the data can be found is required at submission. Your answers to this question constitute the Data Availability Statement and **will be published in the article**, if accepted.<br><br>**Important:** Stating 'data available on request from the author' is not sufficient. If your data are only available upon request, select 'No' for the first question and explain your exceptional situation in the text box.<br><br>Do the authors confirm that all data underlying the findings described in their manuscript are fully available without restriction?	
**Describe where the data may be found in full sentences. If you are copying our sample text, replace any instances of XXX with the appropriate details.**<br><br>• If the data are **held or will be held in a public repository**, include URLs, accession numbers or DOIs. If this information will only be available after acceptance, indicate this by ticking the box below. For example: *All XXX files are available from the XXX database (accession number(s) XXX, XXX.*).<br>• If the data are all contained **within the manuscript and/or Supporting Information files**, enter the following: *All relevant data are within the manuscript and its Supporting Information files.*<br>• If neither of these applies but you are able to provide **details of access elsewhere**, with or without limitations, please do so. For example:<br><br>*Data cannot be shared publicly because of [XXX]. Data are available from the XXX Institutional Data Access / Ethics Committee (contact via XXX) for researchers who meet the criteria for access to confidential data.*<br><br>*The data underlying the results presented in the study are available from (include the name of the third party*	The datasets analyzed during this study are available from the corresponding author on reasonable request. The data are not publicly available due to privacy reasons.

and contact information or URL).<br>• This text is appropriate if the data are owned by a third party and authors do not have permission to share the data.<br><br>* typeset	
Additional data availability information:	

Powered by Editorial Manager® and ProduXion Manager® from Aries Systems Corporation

Manuscript Click here to access/download;Manuscript;Manuscript.docx

Full title: Impact of labor analgesia on mode of delivery and neonatal outcomes in Japan

Short title: Impact of labor analgesia on delivery and neonates

Shu Eguchi[1*], Yuko Nagaoki[1], Sachiko Ohde[2], Michio Hirata, MD[1]

[1]Department of Pediatrics, St. Luke's International Hospital, Tokyo, Japan

[2]Graduate School of Public Health, St. Luke's International University, Tokyo, Japan

*Corresponding author

E-mail: ks0eh1nu0te17@gmail.com (SE)

Abstract

Labor analgesia (LA) is associated with the potential hazard of high-risk delivery, such as cesarean section (CS) and instrumental vaginal delivery (IVD), and adverse neonatal outcomes. Our objective was to examine the impact of LA on mode of delivery and neonatal outcomes and to identify risk factors in pregnant women who should avoid LA. This retrospective cohort study containing 5,184 pregnant women analyzed the association between LA and both mode of delivery and neonatal outcomes. LA increased the risk of IVD (Adjusted Odds Ratio [AOR] 3.25) but decreased that of CS (AOR 0.52). Four factors (advanced maternal age [AOR 1.72], lower maternal height, larger estimated fetal weight, and primipara [AOR 6.76]) increased the risk of IVD. We should carefully consider the indication of LA for cases with these four factors since LA can increase the risk of IVD and adverse neonatal outcomes.

Introduction

Childbirth, as one of the most important life events for most women, requires them to go through a series of burdens during labor and delivery. The magnitude of labor pain is often compared with a finger amputation [1]. Although the pain itself is not life-threatening for pregnant women and infants, it can transiently impair the cognitive function of pregnant women, especially memory function, during early puerperium [2]. Researchers reported that analgesia can relieve these symptoms [3]. Currently, labor analgesia (LA) with various methods is commonly used in modern obstetric practice and includes spinal anesthesia, epidural analgesia, and combined spinal-epidural analgesia [4]. However, the side effects, especially those related to the mode of delivery and neonatal outcomes, are of great concern. Some studies reported that LA is associated with the potential hazards of cesarean section (CS), instrumental vaginal delivery (IVD), and adverse neonatal outcomes [5–11]. While many studies from Europe and the United States concluded that epidural analgesia does not increase the risk of CS or adverse neonatal outcomes [12–14], Seybe et al. indicated that epidural analgesia in Asians may increase the risk of emergency CS (ECS) with an adjusted odds ratio (AOR) of 2.35 (95% confidence interval (95%CI) 1.04–5.34) [5].

The risk of ECS after application of epidural analgesia may vary depending on race,

and thus, a race-specific analysis should be carefully considered. In Japan, where the rate of LA was traditionally significantly low at 6% according to the Japan Society of Obstetrics and Gynecology, compared with 20%–70% in other countries in Europe and the United States, the focus has been on the application of LA in the past 5 years. It is concerning that too few reports on LA in Japanese and Asian pregnant women have been published [15, 16]. Specifically, advanced maternal age is so remarkable in Japan that the maternal age at first birth rose from 25.7 years of age (1975) to 30.7 years of age (2020), according to the Ministry of Health, Labour and Welfare, Japan, and this trend could naturally increase the risk of ECS. A meta-analysis reported by Pinheiro et al. showed that advanced maternal age itself was associated with an increased risk of induced labor and CS [17]. Therefore, it is urgent that data from Japan be analyzed due to the high proportion of advanced maternal age.

We believe that this study, which includes an Asian population with a high rate of advanced maternal age, can contribute to the still controversial knowledge of the risks associated with LA. In our study, we used a retrospective chart review and multivariate analysis to examine the impact of labor analgesia on the mode of delivery and neonatal outcomes at our hospital and aimed to identify the risks of pregnant women who should avoid LA.

Materials and Methods

Study population

We conducted a retrospective cohort study of all pregnant women who were admitted for delivery from April 2017 to August 2021 at St Luke's International Hospital, Tokyo, Japan, where the average number of deliveries is 1,400 per annum. We selected samples of pregnant women based on the inclusion and exclusion criteria shown in Fig 1.

Fig 1. The study patient flow diagram.

* All of these presented with postoperative congenital heart disease.

† Data on labor analgesia due to thrombocytopenia associated with Sjögren's syndrome are unavailable.

Data collection

All data were obtained by chart review from the electronic medical records. Maternal data included age at delivery, which was dichotomized as advanced maternal age, defined as giving birth at age 35 or older [18], or not, height, pre-delivery body weight, pre-delivery body mass index, parity, duration of the second stage of labor, and whether or not LA was applied. Neonatal data included gestational age, gender, Apgar score (1 minute, 5 minute), admission to the neonatal intensive care unit (NICU), neonatal death, which is defined as death within 28 days

of birth, and birth weight. The estimated fetal weight was substituted for the actual birth weight because the estimated fetal weight is not always accurate due to inter-examiner reliability. Pregnant women who received LA for vaginal delivery received either epidural analgesia, combined spinal-epidural analgesia, or dural puncture epidural.

Outcomes and definitions

The primary outcome of our study was the mode of delivery, and the secondary outcomes were neonatal admission to the NICU and Apgar score. The indications for admission to the NICU were gestational age less than 36 weeks, birth weight less than 2,300 g, the occurrence of a respiratory disorder requiring supplemental oxygen or more, and when deemed necessary by neonatologists.

Statistical analysis

When the association between mode of delivery and application of LA was examined, we performed the analysis using two different outcomes related to the mode of delivery: spontaneous vaginal delivery (SVD) and ECS (“SVD versus ECS”) and SVD and IVD (“SVD versus IVD”) with the reasoning that the mode of delivery is often automatically determined by the degree of the fetal head station. When termination is considered, ECS was selected in cases of high fetal head station, while IVD was selected in cases of low fetal head station.

Multivariate logistic regression analysis was performed to calculate the AOR and 95%CI, to examine the impact of LA on the mode of delivery and neonatal outcomes. In addition, we focused only on pregnant women who received LA and attempted to identify the characteristics of patients who should avoid LA. This study was approved by the institutional review board of our institute (Approval code: 21-R113) and was conducted according to the principles of the Declaration of Helsinki. Since this study was an observational retrospective study, the requirement for written informed consent was waived, which allowed us to proceed with our study in an opt-out manner. All data were analyzed using SPSS Statistics (IBM, Armonk, New York, USA). A result was considered statistically significant at $p < 0.05$.

Results

Patient characteristics

The total number of pregnant women who were admitted for delivery was 6,404. After considering inclusion and exclusion criteria, 5,184 women were finally included in the study population (Fig 1). The mean maternal age at delivery was 34.6 years with a standard deviation (SD) of 4.45 (Table 1).

Table 1. Patients characteristics / Labor analgesia group versus Non-labor analgesia group.

	All (N=5184)	Labor analgesia group (N=2607)	Non-labor analgesia group (N=2577)	p-value
Age (year-old)[a]	34.6 (4.45)	34.7 (4.42)	34.6 (4.49)	0.4191
Height (cm)[a]	160 (5.37)	160 (5.26)	160 (5.47)	1
Pre-delivery body weight (kg)[a]	52.4 (7.29)	52.4 (7.12)	52.5 (7.45)	0.6213
Pre-delivery BMI[a]	20.5 (2.62)	20.5 (2.56)	20.5 (2.67)	1
Primiparous[b]	3341 (64.4)	1892 (72.6)	1449 (56.2)	<0.001
Multiparous[b]	1843 (35.6)	715 (27.4)	1128 (43.8)	<0.001
Duration of second stage of labor[a]	12:33 (0:15)	15:38 (0:46)	9:04 (0:13)	<0.001
Mode of delivery[b]				
SVD	3750 (72.3)	1866 (71.6)	1884 (73.1)	0.218
IVD	412 (8.0)	331 (12.7)	81 (3.1)	<0.001
ECS	1022 (19.7)	410 (15.7)	612 (23.7)	<0.001
With labor analgesia[b]	2607 (50.3)	2607 (100)	0 (0.0)	0
Gestational age (weeks)[a]	39.5 (1.2)	39.6 (1.2)	39.4 (1.3)	<0.001
Male[b]	2594 (50.0)	1321 (50.7)	1273 (49.4)	0.359
Birth weight (g)[a]	3084 (377)	3120 (364)	3047 (387)	<0.001
Apgar score[a]				
1 minute	8.04 (0.936)	7.96 (1.05)	8.13 (0.797)	<0.001
5 minute	9.03 (0.603)	9.01 (0.628)	9.05 (0.576)	0.0169
Admission to NICU[b]	350 (6.8)	172 (6.6)	178 (6.9)	0.654
Neonatal death[b]	0 (0.0)	0 (0.0)	0 (0.0)	

[a]Mean (standard deviation), t-test p value.

[b]Number cases (percentage), chi-squared test p value.

BMI = Body mass index, ECS = Emergency cesarean section, IVD = Instrumental vaginal delivery, NICU = Neonatal intensive care unit, SVD = Spontaneous vaginal delivery

The proportion of primipara was higher in the LA group (73%) than in the non-LA group (56%, $p < 0.001$) (Table 1). Primiparous women were more likely to receive LA (57%) than multiparous women (39%).

To examine the association between the mode of delivery and the application or not of LA, a univariate analysis was performed for IVD and ECS against SVD (Table 2). Compared with SVD, both IVD and ECS were associated with a higher proportion of admission to the NICU (IVD: 8.0%, $p = 0.008$ ECS: 12.8%, $p < 0.001$) and lower Apgar score (IVD 1 min/5 min: mean7.63/8.85, SD 1.09/0.603, p < 0.001/< 0.001 ECS 1 min/5 min: mean 7.56/8.82, SD 1.26/0.781, p < 0.001/< 0.001). The rate of LA was significantly higher in IVD (80%, $p < 0.001$) than SVD (50%) and conversely lower in ECS (40%, $p < 0.001$).

Table 2. Mode of delivery.

	SVD (N = 3750)	IVD (N = 412)	ECS (N = 1022)	p-value (SVD versus IVD)	p-value (SVD versus ECS)
Age (year-old)[a]	34.1 (4.36)	35.0 (4.54)	36.0 (4.47)	<0.001	<0.001
Height (cm)[a]	160 (5.27)	160 (5.22)	159 (5.67)	1	<0.001
Pre-delivery body weight (kg)[a]	52.2 (6.92)	51.9 (6.99)	53.5 (8.53)	0.4041	<0.001
Pre-delivery BMI[a]	20.4 (2.44)	20.3 (2.50)	21.2 (3.12)	0.4309	<0.001
Primiparous[b]	2112 (56.3)	361 (87.6)	868 (84.9)	<0.001	<0.001
Multiparous[b]	1638 (43.7)	51 (12.4)	154 (15.1)	<0.001	<0.001
Duration of second stage of labor[a]	11:33 (0:38)	16:12 (0:20)	17:04 (0:19)	<0.001	<0.001
With labor analgesia[b]	1866 (50.0)	331 (80.3)	410 (40.1)	<0.001	<0.001
Gestational age (weeks)[a]	39.4 (1.2)	39.6 (1.2)	39.5 (1.3)	0.0013	0.0204
Male[b]	1797 (47.9)	213 (51.7)	584 (57.1)	0.136	<0.001
Birth weight (g)[a]	3084 (355)	3084 (369)	3078 (451)	1	0.6525
Apgar score[a]					
1 minute	8.22 (0.733)	7.63 (1.09)	7.56 (1.26)	<0.001	<0.001
5 minute	9.11 (0.525)	8.85(0.603)	8.82 (0.781)	<0.001	<0.001
Admission to NICU[b]	186 (5.0)	33 (8.0)	131 (12.8)	0.008	<0.001
Neonatal death[b]	0 (0.0)	0 (0.0)	0 (0.0)		

[a]Mean (standard deviation), t-test p value.

[b]Number cases (percentage), chi-squared test p value.

BMI = Body mass index, ECS = Emergency cesarean section, IVD = Instrumental vaginal delivery, NICU = Neonatal intensive care unit, SVD = Spontaneous vaginal delivery

Primary outcome: Impact of labor analgesia on mode of delivery

First, we extracted the risks that may affect the mode of delivery and then examined the impact of LA on the mode of delivery. We hypothesized that the risks were LA, advanced maternal age, lower maternal height, larger estimated fetal weight, and primipara. The primary outcome for mode of delivery is shown in Table 3. We analyzed two groups: one compared SVD and ECS, excluding IVD (“SVD versus ECS”), and the other compared SVD and IVD, excluding ECS

("SVD versus IVD").

Table 3. The risk of instrumental vaginal delivery and emergency cesarean section, and adverse neonatal outcomes

	Adjusted Odds Ratios	95% Confidence interval	p-value
**ECS (versus SVD)**			
With labor analgesia	0.52	0.44 - 0.60	<0.001
Advanced maternal age (≥ 35 year-old)	2.39	2.05 - 2.78	<0.001
Height	0.95	0.94 - 0.97	<0.001
Estimated fetal weight	1.00	1.00 - 1.00	0.038
Primiparous	6.00	4.95 - 7.28	<0.001
**IVD (versus SVD)**			
With labor analgesia	3.25	2.51 - 4.20	<0.001
Advanced maternal age (≥ 35 year-old)	1.66	1.33 - 2.05	<0.001
Height	0.99	0.97 - 1.01	0.386
Estimated fetal weight	1.00	1.00 - 1.00	0.741
Primiparous	5.13	3.77 - 6.99	<0.001
**IVD (versus SVD) among patients with labor analgesia**			
Advanced maternal age (≥ 35 year-old)	1.72	1.43 - 2.06	<0.001
Height	0.96	0.94 - 0.98	<0.001
Estimated fetal weight	1.00	1.00 - 1.00	<0.001
Primiparous	6.76	5.10 - 8.96	<0.001
**Apgar score 1-minute <7**			
With labor analgesia	2.06	1.50 - 2.83	<0.001
Advanced maternal age (≥ 35 year-old)	1.29	0.96 - 1.74	0.088
Height	0.99	0.96 - 1.02	0.379
Estimated fetal weight	1.00	1.00 - 1.00	0.452
Primiparous	2.68	1.80 - 3.9	<0.001
**Apgar score 5-minute <7**			
With labor analgesia	1.13	0.56 - 2.27	0.741
Advanced maternal age (≥ 35 year-old)	1.53	0.76 - 3.09	0.234
Height	0.92	0.86 - 0.99	0.015
Estimated fetal weight	1.00	1.00 - 1.00	0.227
Primiparous	2.07	0.88 - 4.87	0.096
**Admission to NICU**			
With labor analgesia	1.06	0.85 - 1.33	0.615
Advanced maternal age (≥ 35 year-old)	1.34	1.07 - 1.68	0.011
Height	1.01	0.99 - 1.03	0.338
Estimated fetal weight	1.00	1.00 - 1.00	<0.001
Primiparous	1.68	1.30 - 2.18	<0.001

ECS = Emergency cesarean section, IVD = Instrumental vaginal delivery, NICU = Neonatal

intensive care unit, SVD = Spontaneous vaginal delivery

In "SVD versus ECS," those of advanced maternal age and primipara exhibited a higher AOR (AOR 2.39, 6.00 95%CI 2.05–2.78, 4.95–7.28). Lower maternal height and larger estimated fetal weight increased the risks of ECS, while the use of LA decreased the risk of ECS (AOR 0.52, 95%CI 0.44–0.60).

Similar to the "SVD versus ECS" results, advanced maternal age and primipara also exhibited a higher AOR (AOR 1.66, 5.13 95%CI 1.33–2.05, 3.77–6.99) in the "SVD versus IVD" comparison. Maternal height and estimated fetal weight were not associated with IVD, but the use of LA increased the risk of IVD (AOR 3.25, 95%CI 2.51–4.20).

Secondary outcome: Impact of labor analgesia on neonatal outcomes

The secondary outcomes, including neonatal outcomes and Apgar score, are shown in Table 3. LA lowered the Apgar score at 1 minute (AOR 2.06, 95%CI 1.50–2.83), but it did not influence the Apgar score at 5 minutes or the NICU admission rate. Being a primipara was the strongest factor that could cause all adverse neonatal outcomes after adjusting for covariates including LA prescription.

When the population was restricted to only those who received LA, we found that pregnant women with the following four risk factors demonstrated a higher risk of IVD: advanced maternal age, lower maternal height, larger estimated fetal weight, and primipara (Table 3). The AOR of primipara was especially high at 6.76 (95%CI 5.10–8.96) followed by that of advanced maternal age (AOR 1.72, 95%CI 1.43–2.06). Both lower maternal height and larger estimated fetal weight were significantly associated with an increased risk of IVD ($p < 0.001$).

Discussion

This study included more than 5,000 pregnant women, approximately half of whom were of advanced maternal age, and who were admitted to our hospital for delivery from 2017 to 2021. In a multivariate analysis, LA increased the risk of IVD but decreased that of ECS. Moreover, advanced maternal age and primipara increased the risks of both ECS and IVD. Among those who received LA, all four factors of advanced maternal age, lower maternal height, larger estimated fetal weight, and primipara increased the risk of IVD; the AOR of primipara was highest at 6.76 (95%CI 5.10–8.96) and that of advanced maternal age was second highest at 1.72 (95%CI 1.43–2.06).

To the best of our knowledge, our study is one of few reports from Japan that presents an association between LA and mode of delivery as well as neonatal outcomes. The novelty is that we sought to identify the characteristics of pregnant women who may need to be cautious in selecting LA. Our study is valuable in that we analyzed primarily Asian patients, who are recognized to be at an increased risk of CS [5]; we also included a large number of patients of advanced maternal age.

As our results showed that LA decreased the risk of CS, many recent reports showed that LA is not associated with increased risk of CS or that LA even reduces CS rates [12, 14].

Despite a previous study published several decades ago, which concluded that epidural analgesia was associated with a significantly increased risk of CS among primipara [5], a recent Cochrane Review in 2018 that included 40 trials and more than 11,000 pregnant women found that epidural analgesia did not increase the risk of CS [13]. The reason that LA is not associated with and does not increase the ECS rate may be because factors associated with more painful labor are themselves associated with an increased risk of CS (e.g., fetal malrotation, fetal-pelvic disproportion, dysfunctional labor) [19]. Thus, we consider that pain relief might help to avoid ECS. However, no studies compared the degree of pain with the CS rate, and it is unclear why LA lowers the CS rate. Further investigation is needed to determine the pathophysiological reasons.

Our results showed that LA also increases the risk of IVD. While some studies reported that LA is not associated with the increased risk of IVD [14], many other studies, including a systematic review by Lieberman et al. [7], reported the association between LA and an increased risk of IVD [6, 8, 9, 12]. Lieberman et al. estimated a pooled odds ratio of 2.2 (95%CI 1.3–7.8) [7], which is comparable to the result of our study (AOR 3.25, 95%CI 2.51–4.20), and considered that the most common reason why pregnant women who receive LA are likely to undergo IVD is due to stalled labor, followed by risk to fetal well-being [7].

We found that the use of LA is associated with adverse neonatal outcomes. The effects of LA on neonatal outcomes are controversial and are the same as those on mode of delivery. Although a meta-analysis reported in the Cochrane Review in 2018 denied an association between epidural analgesia and adverse neonatal outcomes, such as admission rate to the NICU and an Apgar score of less than 7 at 5 minutes [13], many other studies confirmed the association [6, 10, 11]. In general, Apgar scores are lower in newborns delivered by CS [21, 22], but in our study, the Apgar score was lower in the LA group even though the CS rate decreased, which suggests that LA itself must lower the Apgar score. Kumar et al. reported that the Apgar score was significantly lower with use of epidural analgesia because of respiratory distress, and they considered that a large volume of distribution at a steady state in neonates resulted in a much longer terminal elimination half-life of fentanyl over several hours [10]. Most newborns with neonatal asphyxia are able to establish fetal-to-neonatal transition after appropriate resuscitation immediately after birth. This means that even if the Apgar score at 1 minute is low, the Apgar score at 5 minutes will be higher after appropriate resuscitation. This may be the reason why the results of our study did not reveal statistically significant differences when the Apgar score at 5 minutes was less than 7. Researchers suggested that LA may elicit more severe adverse effects in a newborn in the immediate postnatal period than what the Apgar

score at 1 minute purportedly represents. Neonatologists are expected to exhibit resuscitation skills for cases of neonatal asphyxia, especially in cases of LA. To promote resuscitation skills, each country, region, and hospital should introduce a certification program for resuscitation skills and provide a training and educational system to maintain higher skills and knowledge. Some medical personnel are qualified but do not engage in resuscitation of newborns in practice. Even if neonatal resuscitation is learned through simulation, this skill is difficult to maintain without practical experience, and thus, aggressive resuscitation practices are important. The centralization of facilities where LA is available might ensure safe deliveries. We should carefully consider LA and limit LA to pregnant women without these risk factors, especially in facilities without personnel, such as pediatricians, neonatologists, and midwives, who attend deliveries.

According to our results, LA may reduce the risk of ECS but increase the risk of IVD and lower the Apgar score at 1 minute. In particular, the increased risk of IVD was significantly higher when the pregnant women were primiparous, of advanced maternal age, short in height, and presented with larger fetal weight were administered LA. While the decrease in ECS is favorable in terms of surgical invasiveness and the safety of the next delivery, the increase in IVD is a serious issue. IVD is associated with many potential risks to both the mother (e.g.,

perineal and vaginal tears) and the newborn (e.g., brachial plexus injury with shoulder dystocia, subgaleal and intracranial hemorrhage) [20]. Prior et al. reported that the Apgar score was lower in infants delivered by IVD than in those delivered by CS [21]. Based on our results, we suggest that pregnant women at an increased risk of IVD, such as those of advanced maternal age (i.e., 35 years of age or older), lower maternal height, larger estimated fetal weight, and primipara, should carefully consider LA. Hence, when obstetricians and/or anesthesiologists confirm a pregnant woman's requests to receive LA, explaining not only the advantages but also the disadvantages and risks and obtaining appropriate informed consent is important. Moreover, pregnant women with these risk factors may be guided to receive alternative treatments, such as acupuncture, hypnotism, yoga, exercise during pregnancy, hydrotherapy, transcutaneous electronic nerve stimulation, massage, and relaxation techniques [4]. Among IVD techniques, obstetric forceps are particularly dangerous in the hands of inexperienced obstetricians. Therefore, LA should be administered only in facilities with well-trained and experienced obstetricians because pregnant women who receive LA are more likely to require IVD.

The results of our study are based on a limited population, and as mentioned above, we should be cautious about perceptions of the indications for and the risks of LA. Determining the indications for LA on a case-by-case basis and recognizing that LA is not beneficial for

every pregnant woman is necessary because the population of pregnant women and facilities vary depending on the country, region, and other circumstances. The environment surrounding childbirth is changing; for example, advanced maternal age is increasing in all developed countries due to women's career advancement and lifestyle changes, and thus, we need to consistently update our research findings on the impacts of LA. In addition, the medical principle behind lower Apgar scores is not completely understood, and since the topic of LA remains controversial, further research is needed.

Our study also exhibits some limitations. The impact of the method of LA on the mode of delivery was not considered. In Tables 1 and 2, although statistically significant differences were observed in terms of maternal age, height, pre-delivery body weight, pre-delivery body mass index, gestational age, sex of newborn, and birth weight, these differences are not noteworthy in clinical practice because these factors were continuous variables and the number of cases that met the inclusion criteria was high. Similarly, in the multivariate analysis, we should focus on the interpretation of the comparison of the AOR of continuous variables, such as maternal height and estimated birth weight, and that of categorical variables, such as LA, advanced maternal age, and primipara. In addition, establishing the cut-off for maternal height and estimated birth weight is very difficult, and thus, the results of our study are unlikely

to affect actual practice without specific values.

In conclusion, although LA is an important means to relieve pain during labor and delivery, it can increase the risk of IVD and an Apgar score of less than 7 at 1 minute. Therefore, we should carefully consider the indication of LA in cases of advanced maternal age, lower maternal height, larger estimated fetal weight, and primipara. Furthermore, it is desirable to maintain and improve resuscitation skills through training and certification systems and to intensify facilities where LA is available.

Acknowledgements

Not applicable.

References

1. Melzack R. The myth of painless childbirth (the John J. Bonica lecture). Pain 1984;19:321–337.
2. Eidelman AI, Hoffmann NW, Kaitz M. Cognitive deficits in women after child-birth. Obstet Gynecol 1993;81:764–767.
3. Hawkins JL. Epidural analgesia for labor and delivery. N Engl J Med 2010;362:1503–1510.

4. Koyyalamudi V, Sidhu G, Cornett EM, Nguyen V, Labrie-Brown C, Fox CJ, et al. New labor pain treatment options. Curr Pain Headache Rep 2016;20:11.
5. Seyb ST, Berka RJ, Socol ML, Dooley SL. Risk of cesarean delivery with elective induction of labor at term in nulliparous women. Obstet Gynecol 1999;94:600–607.
6. Hincz P, Podciechowskil L, Grzesiak M, Horzelski W, Wilczyflski J. Epidural analgesia during labor:a retrospective cohort study on its effects on labour, delivery and neonatal outcome. Ginekol Pol 2014;85:923–928.
7. Lieberman E, O’donoghue C. Unintended effects of epidural analgesia during labor: a systematic review. Am J Obstet Gynecol 2002;186:S31–68.
8. Lim G, Facco FL, Nathan N, Waters JH, Wong CA, Eltzschig HK. A review of the impact of obstetric anesthesia on maternal and neonatal outcomes. Anesthesiology 2018;129:192–215.
9. Herrera-Gómez A, Luna-Bertos ED, Ramos-Torrecillas J, Ocaña-Peinado FM, Ruiz C, García-Martínez O. Risk assessments of epidural analgesia during labor and delivery. Clin Nurs Res 2018;27:841–852.
10. Kumar M, Chandra S, Ijaz Z, Senthilselvan A. Epidural analgesia in labour and neonatal respiratory distress: a case-control study. Arch Dis Child Fetal Neonatal Ed 2014;99:116–

119.

11. Greenwell EA, Wyshak G, Ringer SA, Johnson LC, Rivkin MJ, Lieberman E. Intrapartum temperature elevation, epidural use, and adverse outcome in term infants. Pediatrics 2012;129:e447–454.

12. Schrock SD, Harraway-Smith C. Labor analgesia. Am Fam Physician 2012;85:447–454.

13. Anim-Somuah M, Smyth RM, Cyna AM, Cuthbert A. Epidural versus non-epidural or no analgesia for pain management in labour. Cochrane Database Syst Rev 2018;5:CD000331.

14. Zhang J, Yancey MK, Klebanoff MA, Schwarz J, Schweitzer D. Does epidural analgesia prolong labor and increase risk of cesarean delivery? A natural experiment. Am J Obstet Gynecol 2001;185:128–134.

15. Naito Y, Ida M, Yamamoto R, Tachibana K, Kinouchi K. The effect of labor epidural analgesia on labor, delivery, and neonatal outcomes: a propensity score-matched analysis in a single Japanese institute. JA Clin Rep 2019;5:40.

16. Hu LQ, Zhang J, Wong CA, Cao Q, Zhang G, Rong H, et.al Impact of the introduction of neuraxial labor analgesia on mode of delivery at an urban maternity hospital in China. Int J Gynaecol Obstet 2015;129:17–21.

17. Pinheiro RL, Areia AL, Pinto AM, Donato H. Advanced maternal age: adverse outcomes

of pregnancy, a meta-analysis. Acta Med Port 2019;32:219–226.

18. Dekker R, Breakey AA. Evidence on advanced maternal age. Evidence Based Birth 2016.
19. Wong CA. (2014). Epidural and spinal analgesia/Anesthesia for labor and vaginal delivery, Obstetric Anesthesia: Principles and Practice. In: Chestnut DH (ed). Anesthesiology. Mosby: Maryland Heights, 2014, pp 490.
20. Keriakos R, Sugumar S, Hilal N. Instrumental vaginal delivery--back to basics. J Obstet Gynaecol 2013;33:781–786.
21. Prior T, Kumar S. Mode of delivery has an independent impact on neonatal condition at birth. Eur J Obstet Gynecol Reprod Biol 2014;181:135–139.

Thavarajah H, Flatley C, Kumar S. The relationship between the five minute Apgar score, mode of birth and neonatal outcomes. J Matern Fetal Neonatal Med 2018;31:1335–1341.

Ethics approval and informed consent:

This study was approved by the institutional review board of our institute (Approval code: 21-R113) and was conducted according to the principles of the Declaration of Helsinki.

Consent for publication:

Since this study was an observational retrospective study, written informed consent was waived and we proceeded using an opt-out approach.

Availability of Data and Materials:

The datasets analyzed during this study are available from the corresponding author on reasonable request.

Author Contributions:

All authors contributed to the study conception and design. Material preparation, data collection, and literature review were performed by SE. SE and SO performed data analysis. SE, YN, and SO wrote the original draft of the manuscript. MH supervised the study, including data analysis and interpretation. All authors commented on the previous version of the manuscript and

approved the final manuscript.

Figure 1 Click here to access/download;Figure;Figure 1.tiff

Figure. 1

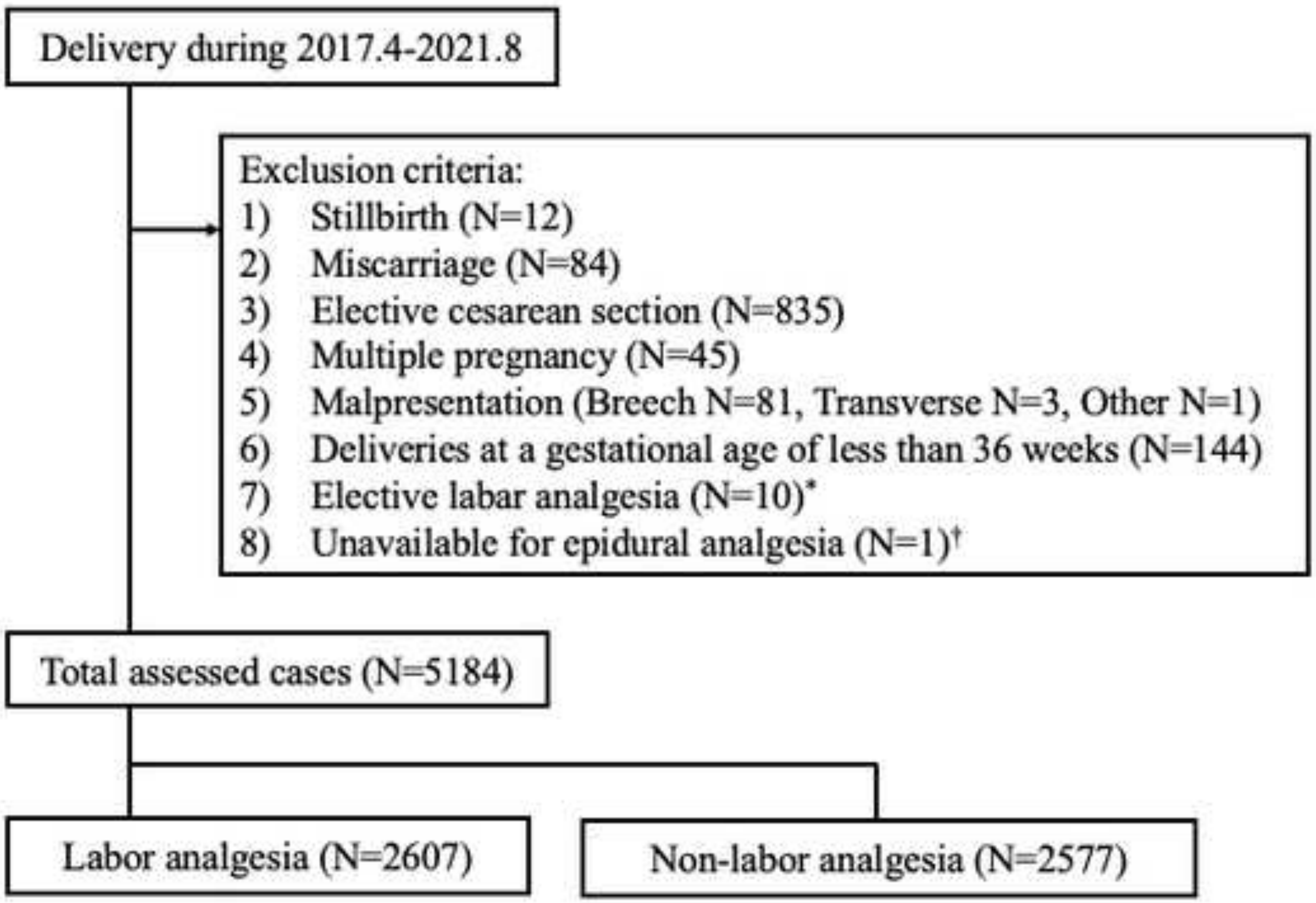

Supplement: Attachment — Submitted filename: PONE-D-22-18968_reviewed.pdf [file pone.0284368.s002.pdf]
