# Supplementary material for: Impact of labor analgesia on mode of delivery and neonatal outcomes in Japan: A retrospective cohort study
Source: PLoS One. 2023 Apr 12;18(4):e0284368. doi: 10.1371/journal.pone.0284368 (PMC10096281; doi:10.1371/journal.pone.0284368)
Supplement: Attachment — Submitted filename: PONE-D-22-18968_R1_reviewed.pdf [file pone.0284368.s004.pdf]

PLOS ONE

Impact of labor analgesia on mode of delivery and neonatal outcomes in Japan: A retrospective cohort study

--Manuscript Draft--

**Manuscript Number:**	PONE-D-22-18968R1
**Article Type:**	Research Article
**Full Title:**	Impact of labor analgesia on mode of delivery and neonatal outcomes in Japan: A retrospective cohort study
**Short Title:**	Impact of labor analgesia on delivery and neonates
**Corresponding Author:**	Shu Eguchi<br>Saint Luke's International Hospital: Sei Roka Kokusai Byoin<br>Tokyo, JAPAN
**Keywords:**	labor analgesia; cesarean section; instrumental vaginal delivery; Neonate
**Abstract:**	Labor analgesia (LA) is associated with the potential hazard of high-risk delivery, such as cesarean section (CS) and instrumental vaginal delivery (IVD), and adverse neonatal outcomes such as neonatal asphyxia and respiratory distress. Our objective was to examine the impact of LA on mode of delivery and neonatal outcomes and to counsel pregnant women about a potentially higher risk and allow them to decide LA or non-LA. This retrospective cohort study containing 5,184 pregnant women analyzed the association between LA and both mode of delivery and neonatal outcomes. LA increased the risk of IVD (Adjusted Odds Ratio [AOR] 3.25, 95% confidence interval [95%CI] 2.51–4.20) but decreased that of CS (AOR 0.52, 95%CI 0.44–0.60). Two factors (advanced maternal age [AOR 1.70, 95%CI 1.33–2.17] and primipara [AOR 4.72, 95%CI 3.30–6.75]) increased the risk of IVD. We should carefully consider the indication of LA for cases with these two factors since LA can increase the risk of IVD and adverse neonatal outcomes.
**Order of Authors:**	Shu Eguchi
	Yuko Nagaoki
	Sachiko Ohde
	Michio Hirata
**Response to Reviewers:**	Dear Emily Chenette, Editor-in-Chief<br><br>I don't think I have deviated from the submission guideline, but I have received some pointers from Reviewer #6. I would appreciate any advice you could give me.
**Additional Information:**	
**Question**	**Response**
**Financial Disclosure**<br><br>Enter a financial disclosure statement that describes the sources of funding for the work included in this submission. Review the submission guidelines for detailed requirements. View published research articles from *PLOS ONE* for specific examples.<br><br>This statement is required for submission and **will appear in the published article** if the submission is accepted. Please make sure it is accurate.	Since this study was an observational retrospective study, written informed consent was waived and we proceeded using an opt-out approach. This study received no specific grant from any funding agency in the public, commercial, or not-for-profit sectors.

Powered by Editorial Manager® and ProduXion Manager® from Aries Systems Corporation

**Unfunded studies**<br>Enter: *The author(s) received no specific funding for this work.*<br><br>**Funded studies**<br>Enter a statement with the following details:<br>• Initials of the authors who received each award<br>• Grant numbers awarded to each author<br>• The full name of each funder<br>• URL of each funder website<br>• Did the sponsors or funders play any role in the study design, data collection and analysis, decision to publish, or preparation of the manuscript?<br>• **NO** - Include this sentence at the end of your statement: *The funders had no role in study design, data collection and analysis, decision to publish, or preparation of the manuscript.*<br>• **YES** - Specify the role(s) played.<br><br>* typeset	
**Competing Interests**<br><br>Use the instructions below to enter a competing interest statement for this submission. On behalf of all authors, disclose any competing interests that could be perceived to bias this work—acknowledging all financial support and any other relevant financial or non-financial competing interests.<br><br>This statement is required for submission and **will appear in the published article** if the submission is accepted. Please make sure it is accurate and that any funding sources listed in your Funding Information later in the submission form are also declared in your Financial Disclosure statement.<br><br>View published research articles from *PLOS ONE* for specific examples.	The authors have declared that no competing interests exist.

**NO authors have competing interests**<br><br>Enter: *The authors have declared that no competing interests exist.*<br><br>**Authors with competing interests**<br><br>Enter competing interest details beginning with this statement:<br><br>*I have read the journal's policy and the authors of this manuscript have the following competing interests: [insert competing interests here]*<br><br>* typeset	
**Ethics Statement**<br><br>Enter an ethics statement for this submission. This statement is required if the study involved:<br><br>• Human participants<br>• Human specimens or tissue<br>• Vertebrate animals or cephalopods<br>• Vertebrate embryos or tissues<br>• Field research<br><br>Write "N/A" if the submission does not require an ethics statement.<br><br>General guidance is provided below. Consult the submission guidelines for detailed instructions. **Make sure that all information entered here is included in the Methods section of the manuscript.**	This study was approved by the institutional review board of our institute (Approval code: 21-R113) and was conducted according to the principles of the Declaration of Helsinki.<br><br>Since this study was an observational retrospective study, written informed consent was waived and we proceeded using an opt-out approach.

**Format for specific study types**<br><br>**Human Subject Research (involving human participants and/or tissue)**<br>• Give the name of the institutional review board or ethics committee that approved the study<br>• Include the approval number and/or a statement indicating approval of this research<br>• Indicate the form of consent obtained (written/oral) or the reason that consent was not obtained (e.g. the data were analyzed anonymously)<br><br>**Animal Research (involving vertebrate animals, embryos or tissues)**<br>• Provide the name of the Institutional Animal Care and Use Committee (IACUC) or other relevant ethics board that reviewed the study protocol, and indicate whether they approved this research or granted a formal waiver of ethical approval<br>• Include an approval number if one was obtained<br>• If the study involved *non-human primates*, add *additional details* about animal welfare and steps taken to ameliorate suffering<br>• If anesthesia, euthanasia, or any kind of animal sacrifice is part of the study, include briefly which substances and/or methods were applied<br><br>**Field Research**<br><br>Include the following details if this study involves the collection of plant, animal, or other materials from a natural setting:<br>• Field permit number<br>• Name of the institution or relevant body that granted permission	
**Data Availability**<br><br>Authors are required to make all data underlying the findings described fully available, without restriction, and from the time of publication. PLOS allows rare exceptions to address legal and ethical concerns. See the PLOS Data Policy and FAQ for detailed information.	No - some restrictions will apply

A Data Availability Statement describing where the data can be found is required at submission. Your answers to this question constitute the Data Availability Statement and **will be published in the article**, if accepted.<br><br>**Important:** Stating 'data available on request from the author' is not sufficient. If your data are only available upon request, select 'No' for the first question and explain your exceptional situation in the text box.<br><br>Do the authors confirm that all data underlying the findings described in their manuscript are fully available without restriction?	
**Describe where the data may be found in full sentences. If you are copying our sample text, replace any instances of XXX with the appropriate details.**<br><br>• If the data are **held or will be held in a public repository**, include URLs, accession numbers or DOIs. If this information will only be available after acceptance, indicate this by ticking the box below. For example: *All XXX files are available from the XXX database (accession number(s) XXX, XXX.*).<br>• If the data are all contained **within the manuscript and/or Supporting Information files**, enter the following: *All relevant data are within the manuscript and its Supporting Information files.*<br>• If neither of these applies but you are able to provide **details of access elsewhere**, with or without limitations, please do so. For example:<br><br>*Data cannot be shared publicly because of [XXX]. Data are available from the XXX Institutional Data Access / Ethics Committee (contact via XXX) for researchers who meet the criteria for access to confidential data.*<br><br>*The data underlying the results presented in the study are available from (include the name of the third party*	Data cannot be shared publicly because of the law of St. Luke's International Hospital institutional review board. Data are available from the Research Support Unit of St. Luke's International University for researchers who meet the criteria for access to confidential data. The address for contact office of the Research Support Unit of St. Luke's International University is as follows e-mail: kenkyukikaku@luke.ac.jp

and contact information or URL).<br>• This text is appropriate if the data are owned by a third party and authors do not have permission to share the data.<br><br>* typeset	
Additional data availability information:	

Powered by Editorial Manager® and ProduXion Manager® from Aries Systems Corporation

Manuscript
Click here to access/download;Manuscript;Manuscript.docx

Full title: Impact of labor analgesia on mode of delivery and neonatal outcomes in Japan: A retrospective cohort study.

Short title: Impact of labor analgesia on delivery and neonates

Shu Eguchi[1*], Yuko Nagaoki[1], Sachiko Ohde[2], Michio Hirata, MD[1]

[1]Department of Pediatrics, St. Luke's International Hospital, Tokyo, Japan

[2]Graduate School of Public Health, St. Luke's International University, Tokyo, Japan

*Corresponding author

E-mail: ks0eh1nu0te17@gmail.com (SE)

Abstract

Labor analgesia (LA) is associated with the potential hazard of high-risk delivery, such as cesarean section (CS) and instrumental vaginal delivery (IVD), and adverse neonatal outcomes such as neonatal asphyxia and respiratory distress. Our objective was to examine the impact of LA on mode of delivery and neonatal outcomes and to counsel pregnant women about a potentially higher risk and allow them to decide LA or non-LA. This retrospective cohort study containing 5,184 pregnant women analyzed the association between LA and both mode of delivery and neonatal outcomes. LA increased the risk of IVD (Adjusted Odds Ratio [AOR] 3.25, 95% confidence interval [95%CI] 2.51–4.20) but decreased that of CS (AOR 0.52, 95%CI 0.44–0.60). Two factors (advanced maternal age [AOR 1.70, 95%CI 1.33–2.17] and primipara [AOR 4.72, 95%CI 3.30–6.75]) increased the risk of IVD. We should carefully consider the indication of LA for cases with these two factors since LA can increase the risk of IVD and adverse neonatal outcomes.

Introduction

Childbirth, as one of the most important life events for most women, requires them to go through a series of burdens during labor and delivery. The magnitude of labor pain is often compared with a finger amputation [1]. Although the pain itself is not life-threatening for pregnant women and infants, it can transiently impair the cognitive function of pregnant women, especially memory function, during early puerperium [2]. Furthermore, the pain is related to the risk of postpartum depression, and the degree of pain is also associated with the severity of depression [3]. Researchers reported that analgesia can relieve these symptoms [4]. Currently, labor analgesia (LA) with various methods is commonly used in modern obstetric practice and includes spinal anesthesia, epidural analgesia, and combined spinal-epidural analgesia [5]. However, the side effects, especially those related to the mode of delivery and neonatal outcomes, are of great concern. Some studies reported that LA is associated with the potential hazards of cesarean section (CS), instrumental vaginal delivery (IVD), and adverse neonatal outcomes [6–12]. While many studies from Europe and the United States concluded that epidural analgesia does not increase the risk of CS or adverse neonatal outcomes [13–15], Seybe et al. indicated that epidural analgesia in Asians may increase the risk of emergency CS (ECS) with an adjusted odds ratio (AOR) of 2.35 (95% confidence interval [95%CI] 1.04–5.34) [6].

The risk of ECS after application of epidural analgesia may vary depending on race, and thus, a race-specific analysis should be carefully considered. In Japan, where the rate of LA was traditionally significantly low at 6% according to the Japan Society of Obstetrics and Gynecology, compared with 20%–70% in other countries in Europe and the United States, the focus has been on the application of LA in the past 5 years. It is concerning that too few reports on LA in Japanese and Asian pregnant women have been published [16, 17]. Specifically, advanced maternal age is so remarkable in Japan that the maternal age at first birth rose from 25.7 years of age (1975) to 30.7 years of age (2020), according to the Ministry of Health, Labour and Welfare, Japan, and this trend could naturally increase the risk of ECS. A meta-analysis reported by Pinheiro et al. showed that advanced maternal age itself was associated with an increased risk of induced labor and CS [18]. Therefore, it is urgent that data from Japan be analyzed due to the high proportion of advanced maternal age.

We believe that this study, which includes an Asian population with a high rate of advanced maternal age, can contribute to the still controversial knowledge of the risks associated with LA. In our study, we used a retrospective chart review and multivariate analysis to examine the impact of labor analgesia on the mode of delivery and neonatal outcomes at our hospital and aimed to counsel pregnant women about a potentially higher risk and allow them

to decide LA or non-LA.

Materials and Methods

Study population

We conducted a retrospective cohort study of all pregnant women who were admitted for delivery from April 2017 to August 2021 at St Luke's International Hospital, Tokyo, Japan, where the average number of deliveries is 1,400 per annum. We selected samples of pregnant women based on the inclusion and exclusion criteria shown in Fig 1. We included all pregnant women who were admitted for delivery in the study periods. The exclusion criteria were as follows Stillbirths and miscarriages were excluded. Since a mode of delivery was defined as the primary outcome, cases with elective CS were excluded. For the same reason, cases with multiple pregnancy and malpresentation that would have resulted in elective CS were also excluded. Cases of delivery at a gestational age of less than 36 weeks were excluded because these could affect a mode of delivery and neonatal outcomes. Finally, cases with elective LA, cases in which LA was unavailable for LA, and cases with the lack of data due to outborn were excluded. All of the cases with elective LA had postoperative congenital heart disease, and the attending physician decided to conduct labor analgesia without the patient's wishes. The one patient with thrombocytopenia associated with Sjögren's syndrome who could not receive LA was excluded.

Fig 1. The study patient flow diagram.

Data collection

All data were obtained by chart review from the electronic medical records. Maternal data included age at delivery, which was dichotomized as advanced maternal age, defined as giving birth at age 35 or older [19], or not, height, pre-delivery body weight, pre-delivery body mass index, parity, duration of the second stage of labor, and whether or not LA was applied. Neonatal data included gestational age, gender, Apgar score (1 minute, 5 minute), admission to the neonatal intensive care unit (NICU), neonatal death, which is defined as death within 28 days of birth, and birth weight. The estimated fetal weight was replaced with the actual birth weight because the estimated fetal weight is not always accurate due to inter-examiner reliability. Pregnant women who received LA for vaginal delivery received either epidural analgesia, combined spinal-epidural analgesia, or dural puncture epidural.

Outcomes and definitions

The primary outcome of our study was the mode of delivery, and the secondary outcomes were neonatal admission to the NICU and Apgar score. The indications for admission to the NICU were gestational age less than 36 weeks, birth weight less than 2,300 g, the occurrence of a respiratory disorder requiring supplemental oxygen or more, and when deemed necessary by

neonatologists.

Statistical analysis

When the association between mode of delivery and application of LA was examined, we performed the analysis using two different outcomes related to the mode of delivery: spontaneous vaginal delivery (SVD) and ECS (“SVD versus ECS”) and SVD and IVD (“SVD versus IVD”) with the reasoning that the mode of delivery is often automatically determined by the degree of the fetal head station. When immediate delivery is considered, ECS was selected in cases of high fetal head station, while IVD was selected in cases of low fetal head station. The high fetal head station means at +2 or more. The final decision is made by the attending obstetrician. Multivariate logistic regression analysis was performed to calculate the AOR and 95%CI, to examine the impact of LA on the mode of delivery and neonatal outcomes. In addition, we focused only on pregnant women who received LA and attempted to identify the characteristics of patients with factors that make LA risky. This study was approved by the institutional review board of our institute (Approval code: 21-R113) and was conducted according to the principles of the Declaration of Helsinki. Since this study was an observational retrospective study, the requirement for written informed consent was waived, which allowed us to proceed with our study in an opt-out manner. All data were analyzed using SPSS Statistics

(IBM, Armonk, New York, USA). A result was considered statistically significant at $p < 0.05$.

Results

Patient characteristics

The total number of pregnant women who were admitted for delivery was 6,404. After considering inclusion and exclusion criteria, 5,184 women were finally included in the study population (Fig 1). The mean maternal age at delivery was 34.6 years with a standard deviation (SD) of 4.45 (Table 1).

Table 1. Patients characteristics / Labor analgesia group versus Non-labor analgesia group.

	All (N=5184)	Labor analgesia group (N=2607)	Non-labor analgesia group (N=2577)	p-value
Age (year-old)[a]	34.6 (4.45)	34.7 (4.42)	34.6 (4.49)	0.4191
Height (cm)[a]	160 (5.37)	160 (5.26)	160 (5.47)	1
Pre-delivery body weight (kg)[a]	52.4 (7.29)	52.4 (7.12)	52.5 (7.45)	0.6213
Pre-delivery BMI[a]	20.5 (2.62)	20.5 (2.56)	20.5 (2.67)	1
Primiparous[b]	3341 (64.4)	1892 (72.6)	1449 (56.2)	<0.001
Multiparous[b]	1843 (35.6)	715 (27.4)	1128 (43.8)	<0.001
Duration of second stage of labor[a]	12:33 (0:15)	15:38 (0:46)	9:04 (0:13)	<0.001
Mode of delivery[b]				
SVD	3750 (72.3)	1866 (71.6)	1884 (73.1)	0.218
IVD	412 (8.0)	331 (12.7)	81 (3.1)	<0.001
ECS	1022 (19.7)	410 (15.7)	612 (23.7)	<0.001
With labor analgesia[b]	2607 (50.3)	2607 (100)	0 (0.0)	0
Gestational age (weeks)[a]	39.5 (1.2)	39.6 (1.2)	39.4 (1.3)	<0.001
Male[b]	2594 (50.0)	1321 (50.7)	1273 (49.4)	0.359
Birth weight (g)[a]	3084 (377)	3120 (364)	3047 (387)	<0.001
Apgar score[a]				
1 minute	8.04 (0.936)	7.96 (1.05)	8.13 (0.797)	<0.001
5 minute	9.03 (0.603)	9.01 (0.628)	9.05 (0.576)	0.0169
Admission to NICU[b]	350 (6.8)	172 (6.6)	178 (6.9)	0.654
Neonatal death[b]	0 (0.0)	0 (0.0)	0 (0.0)	

BMI = Body mass index, ECS = Emergency cesarean section, IVD = Instrumental vaginal

delivery, NICU = Neonatal intensive care unit, SVD = Spontaneous vaginal delivery

[a]Mean (standard deviation), t-test p value.

[b]Number cases (percentage), chi-squared test p value.

The proportion of primipara was higher in the LA group (73%) than in the non-LA group (56%, $p < 0.001$) (Table 1). Primiparous women were more likely to receive LA (57%) than multiparous women (39%).

To examine the association between the mode of delivery and the application or not of LA, a univariate analysis was performed for IVD and ECS against SVD (Table 2). Compared with SVD, both IVD and ECS were associated with a higher proportion of admission to the NICU (IVD: 8.0%, $p = 0.008$ ECS: 12.8%, $p < 0.001$) and lower Apgar score (IVD 1 min/5 min: mean7.63/8.85, SD 1.09/0.603, p < 0.001/< 0.001 ECS 1 min/5 min: mean 7.56/8.82, SD 1.26/0.781, p < 0.001/< 0.001). The rate of LA was significantly higher in IVD (80%, $p < 0.001$) than SVD (50%) and conversely lower in ECS (40%, $p < 0.001$).

Table 2. Mode of delivery.

	SVD (N = 3750)	IVD (N = 412)	ECS (N = 1022)	p-value (SVD versus IVD)	p-value (SVD versus ECS)
Age (year-old)[a]	34.1 (4.36)	35.0 (4.54)	36.0 (4.47)	<0.001	<0.001
Height (cm)[a]	160 (5.27)	160 (5.22)	159 (5.67)	1	<0.001
Pre-delivery body weight (kg)[a]	52.2 (6.92)	51.9 (6.99)	53.5 (8.53)	0.4041	<0.001
Pre-delivery BMI[a]	20.4 (2.44)	20.3 (2.50)	21.2 (3.12)	0.4309	<0.001
Primiparous[b]	2112 (56.3)	361 (87.6)	868 (84.9)	<0.001	<0.001
Multiparous[b]	1638 (43.7)	51 (12.4)	154 (15.1)	<0.001	<0.001
Duration of second stage of labor[a]	11:33 (0:38)	16:12 (0:20)	17:04 (0:19)	<0.001	<0.001
With labor analgesia[b]	1866 (50.0)	331 (80.3)	410 (40.1)	<0.001	<0.001
Gestational age (weeks)[a]	39.4 (1.2)	39.6 (1.2)	39.5 (1.3)	0.0013	0.0204
Male[b]	1797 (47.9)	213 (51.7)	584 (57.1)	0.136	<0.001
Birth weight (g)[a]	3084 (355)	3084 (369)	3078 (451)	1	0.6525
Apgar score[a]					
1 minute	8.22 (0.733)	7.63 (1.09)	7.56 (1.26)	<0.001	<0.001
5 minute	9.11 (0.525)	8.85(0.603)	8.82 (0.781)	<0.001	<0.001
Admission to NICU[b]	186 (5.0)	33 (8.0)	131 (12.8)	0.008	<0.001
Neonatal death[b]	0 (0.0)	0 (0.0)	0 (0.0)		

BMI = Body mass index, ECS = Emergency cesarean section, IVD = Instrumental vaginal delivery, NICU = Neonatal intensive care unit, SVD = Spontaneous vaginal delivery

[a]Mean (standard deviation), t-test p value.

[b]Number cases (percentage), chi-squared test p value.

Primary outcome: Impact of labor analgesia on mode of delivery

First, we extracted the risks that may affect the mode of delivery and then examined the impact of LA on the mode of delivery. We hypothesized that the risks were LA, advanced maternal age, lower maternal height, larger estimated fetal weight, and primipara. The primary outcome for mode of delivery is shown in Table 3. We analyzed two groups: one compared SVD and ECS, excluding IVD (“SVD versus ECS”), and the other compared SVD and IVD, excluding ECS

("SVD versus IVD").

Table 3. The risk of instrumental vaginal delivery and emergency cesarean section, and adverse neonatal outcomes

	Adjusted Odds Ratios	95% Confidence interval	p-value
**ECS (versus SVD)**			
With labor analgesia	0.52	0.44 - 0.60	<0.001
Advanced maternal age (≥ 35 year-old)	2.39	2.05 - 2.78	<0.001
Height	0.95	0.94 - 0.97	<0.001
Estimated fetal weight	1.00	1.00 - 1.00	0.038
Primiparous	6.00	4.95 - 7.28	<0.001
**IVD (versus SVD)**			
With labor analgesia	3.25	2.51 - 4.20	<0.001
Advanced maternal age (≥ 35 year-old)	1.66	1.33 - 2.05	<0.001
Height	0.99	0.97 - 1.01	0.386
Estimated fetal weight	1.00	1.00 - 1.00	0.741
Primiparous	5.13	3.77 - 6.99	<0.001
**IVD (versus SVD) among patients with labor analgesia**			
Advanced maternal age (≥ 35 year-old)	1.70	1.33 - 2.17	<0.001
Height	0.98	0.96 - 1.01	0.167
Estimated fetal weight	1.00	1.00 - 1.00	0.643
Primiparous	4.72	3.30 - 6.75	<0.001
**IVD (versus SVD) among patients without labor analgesia**			
Advanced maternal age (≥ 35 year-old)	1.49	0.94 - 2.35	0.093
Height	1.02	0.97 - 1.06	0.481
Estimated fetal weight	1.00	1.00 - 1.00	0.895
Primiparous	6.23	3.37 - 11.52	<0.001
**Apgar score 1-minute <7**			
With labor analgesia	2.06	1.50 - 2.83	<0.001
Advanced maternal age (≥ 35 year-old)	1.29	0.96 - 1.74	0.088
Height	0.99	0.96 - 1.02	0.379
Estimated fetal weight	1.00	1.00 - 1.00	0.452
Primiparous	2.68	1.80 - 3.9	<0.001
**Apgar score 5-minute <7**			
With labor analgesia	1.13	0.56 - 2.27	0.741
Advanced maternal age (≥ 35 year-old)	1.53	0.76 - 3.09	0.234
Height	0.92	0.86 - 0.99	0.015
Estimated fetal weight	1.00	1.00 - 1.00	0.227
Primiparous	2.07	0.88 - 4.87	0.096
**Admission to NICU**			
With labor analgesia	1.06	0.85 - 1.33	0.615
Advanced maternal age (≥ 35 year-old)	1.34	1.07 - 1.68	0.011
Height	1.01	0.99 - 1.03	0.338
Estimated fetal weight	1.00	1.00 - 1.00	<0.001
Primiparous	1.68	1.30 - 2.18	<0.001

ECS = Emergency cesarean section, IVD = Instrumental vaginal delivery, NICU = Neonatal intensive care unit, SVD = Spontaneous vaginal delivery

In "SVD versus ECS," those of advanced maternal age and primipara exhibited a higher AOR (AOR 2.39, 6.00 95%CI 2.05–2.78, 4.95–7.28). Lower maternal height and larger estimated fetal weight increased the risks of ECS, while the use of LA decreased the risk of ECS (AOR 0.52, 95%CI 0.44–0.60).

Similar to the "SVD versus ECS" results, advanced maternal age and primipara also exhibited a higher AOR (AOR 1.66, 5.13 95%CI 1.33–2.05, 3.77–6.99) in the "SVD versus IVD" comparison. Maternal height and estimated fetal weight were not associated with the increased risk of IVD, but the use of LA increased the risk of IVD (AOR 3.25, 95%CI 2.51–4.20).

Secondary outcome: Impact of labor analgesia on neonatal outcomes

The secondary outcomes, including neonatal outcomes and Apgar score, are shown in Table 3. LA lowered the Apgar score at 1 minute (AOR 2.06, 95%CI 1.50–2.83), but it did not influence the Apgar score at 5 minutes or the NICU admission rate. Being a primipara was the strongest

factor that could cause all adverse neonatal outcomes after adjusting for covariates including LA prescription.

When the population was restricted to only those who received LA, we found that pregnant women with the following two risk factors demonstrated a higher risk of IVD: advanced maternal age (AOR 1.70, 95%CI 1.33–2.17) and primipara (AOR 4.72, 95%CI 3.30–6.75) (Table 3). Neither lower maternal height nor larger estimated fetal weight were significantly associated with an increased risk of IVD .

Discussion

This study included more than 5,000 pregnant women, approximately half of whom were of advanced maternal age, and who were admitted to our hospital for delivery from 2017 to 2021. In a multivariate analysis, LA increased the risk of IVD but decreased that of ECS. Moreover, advanced maternal age and primipara increased the risks of both ECS and IVD. Among those who received LA, two factors of advanced maternal age and primipara increased the risk of IVD; the AOR of primipara was highest at 4,72 (95%CI 3.30–6.75) and that of advanced maternal age was 1.70 (95%CI 1.33–2.17).

To the best of our knowledge, our study is one of few reports from Japan that presents an association between LA and mode of delivery as well as neonatal outcomes. The novelty is that we sought to identify the characteristics of pregnant women who may need to be cautious in selecting LA. Our study is valuable in that we analyzed primarily Asian patients, who are recognized to be at an increased risk of CS [6]; we also included a large number of patients of advanced maternal age.

As our results showed that LA decreased the risk of CS, many recent reports showed that LA is not associated with increased risk of CS or that LA even reduces CS rates [13, 15]. Despite a previous study published several decades ago, which concluded that epidural

analgesia was associated with a significantly increased risk of CS among primipara [6], a recent Cochrane Review in 2018 that included 40 trials and more than 11,000 pregnant women found that epidural analgesia did not increase the risk of CS [14]. The reason that LA is not associated with and does not increase the ECS rate may be because factors associated with more painful labor are themselves associated with an increased risk of CS (e.g., fetal malrotation, fetal-pelvic disproportion, dysfunctional labor) [20]. Thus, we consider that pain relief might help to avoid ECS. However, no studies compared the degree of pain with the CS rate, and it is unclear why LA lowers the CS rate. Further investigation is needed to determine the pathophysiological reasons.

Our results showed that LA also increases the risk of IVD. While some studies reported that LA is not associated with the increased risk of IVD [15], many other studies, including a systematic review by Lieberman et al. [8], reported the association between LA and an increased risk of IVD [7, 9, 10, 13]. Lieberman et al. estimated a pooled odds ratio of 2.2 (95%CI 1.3–7.8) [8], which is comparable to the result of our study (AOR 3.25, 95%CI 2.51–4.20), and considered that the most common reason why pregnant women who receive LA are likely to undergo IVD is due to stalled labor, followed by risk to fetal well-being [8].

We found that the use of LA is associated with adverse neonatal outcomes. The effects

of LA on neonatal outcomes are controversial and are the same as those on mode of delivery. Although a meta-analysis reported in the Cochrane Review in 2018 denied an association between epidural analgesia and adverse neonatal outcomes, such as admission rate to the NICU and an Apgar score of less than 7 at 5 minutes [14], many other studies confirmed the association [7, 11, 12]. In general, Apgar scores are lower in newborns delivered by CS [22, 23], but in our study, the Apgar score was lower in the LA group even though the CS rate decreased, which suggests that LA itself must lower the Apgar score. Kumar et al. reported that the Apgar score was significantly lower with use of epidural analgesia because of respiratory distress, and they considered that a large volume of distribution at a steady state in neonates resulted in a much longer terminal elimination half-life of fentanyl over several hours [11]. Most newborns with neonatal asphyxia are able to establish fetal-to-neonatal transition after appropriate resuscitation immediately after birth. This means that even if the Apgar score at 1 minute is low, the Apgar score at 5 minutes will be higher after appropriate resuscitation. This may be the reason why the results of our study did not reveal statistically significant differences when the Apgar score at 5 minutes was less than 7. Researchers suggested that LA may elicit more severe adverse effects in a newborn in the immediate postnatal period than what the Apgar score at 1 minute purportedly represents. Neonatologists are expected to exhibit resuscitation

skills for cases of neonatal asphyxia, especially in cases of LA. To promote resuscitation skills, we should provide a training and educational system to maintain higher skills and knowledge. Even if neonatal resuscitation is learned through simulation, this skill is difficult to maintain without practical experience, and thus, aggressive resuscitation practices are important. The centralization of facilities where LA is available might ensure safe deliveries. We should carefully consider LA, especially in facilities without personnel, such as pediatricians, neonatologists, and midwives, who attend deliveries.

According to our results, LA may reduce the risk of ECS but increase the risk of IVD and lower the Apgar score at 1 minute. In particular, the increased risk of IVD was significantly higher when the pregnant women who were primiparous and of advanced maternal age were administered LA. While the decrease in ECS is favorable in terms of surgical invasiveness and the safety of the next delivery, the increase in IVD is a serious issue. IVD is associated with many potential risks to both the mother (e.g., perineal and vaginal tears) and the newborn (e.g., brachial plexus injury with shoulder dystocia, subgaleal and intracranial hemorrhage) [21]. Prior et al. reported that the Apgar score was lower in infants delivered by IVD than in those delivered by CS [22]. Based on our results, we suggest that pregnant women at an increased risk of IVD, such as those of advanced maternal age (i.e., 35 years of age or older), lower

maternal height, larger estimated fetal weight, and primipara, should carefully consider LA. Hence, when obstetricians and/or anesthesiologists confirm a pregnant woman's requests to receive LA, explaining not only the advantages but also the disadvantages and risks and obtaining appropriate informed consent is important. Moreover, pregnant women with these risk factors may be guided to receive alternative treatments, such as acupuncture, hypnotism, yoga, exercise during pregnancy, hydrotherapy, transcutaneous electronic nerve stimulation, massage, and relaxation techniques [5]. Among IVD techniques, obstetric forceps are particularly dangerous in the hands of inexperienced obstetricians. Therefore, LA should be administered only in facilities with well-trained and experienced obstetricians because pregnant women who receive LA are more likely to require IVD.

The results of our study are based on a limited population, and as mentioned above, we should be cautious about perceptions of the indications for and the risks of LA. Determining the indications for LA on a case-by-case basis and recognizing that LA is not beneficial for every pregnant woman is necessary because the population of pregnant women and facilities vary depending on the country, region, and other circumstances. The environment surrounding childbirth is changing; for example, advanced maternal age is increasing in all developed countries due to women's career advancement and lifestyle changes, and thus, we need to

consistently update our research findings on the impacts of LA. In addition, the medical principle behind lower Apgar scores is not completely understood, and since the topic of LA remains controversial, further research is needed.

Our study also exhibits some limitations. The impact of the method of LA on the mode of delivery was not considered. In Tables 1 and 2, although statistically significant differences were observed in terms of maternal age, height, pre-delivery body weight, pre-delivery body mass index, gestational age, sex of newborn, and birth weight, these differences are not noteworthy in clinical practice because these factors were continuous variables and the number of cases that met the inclusion criteria was high. Similarly, in the multivariate analysis, we should focus on the interpretation of the comparison of the AOR of continuous variables, such as maternal height and estimated birth weight, and that of categorical variables, such as LA, advanced maternal age, and primipara. In addition, establishing the cut-off for maternal height and estimated birth weight is very difficult, and thus, the results of our study are unlikely to affect actual practice without specific values.

In conclusion, although LA is an important means to relieve pain during labor and delivery, it can increase the risk of IVD and an Apgar score of less than 7 at 1 minute. Therefore, we should carefully consider the indication of LA in cases of advanced maternal age and

primipara. Furthermore, it is desirable to maintain and improve resuscitation skills through training and certification systems and to intensify facilities where LA is available.

Acknowledgements

Not applicable.

References

1. Melzack R. The myth of painless childbirth (the John J. Bonica lecture). Pain 1984;19:321–337.
2. Eidelman AI, Hoffmann NW, Kaitz M. Cognitive deficits in women after child-birth. Obstet Gynecol 1993;81:764–767.
3. Eisenach JC, Pan PH, Smiley R, Lavand'homme P, Landau R, Houle TT. Severity of acute pain after childbirth, but not type of delivery, predicts persistent pain and postpartum depression. Pain 2008;140(1):87–94.
4. Hawkins JL. Epidural analgesia for labor and delivery. N Engl J Med 2010;362:1503–1510.
5. Koyyalamudi V, Sidhu G, Cornett EM, Nguyen V, Labrie-Brown C, Fox CJ, et al. New labor pain treatment options. Curr Pain Headache Rep 2016;20:11.

6. Seyb ST, Berka RJ, Socol ML, Dooley SL. Risk of cesarean delivery with elective induction of labor at term in nulliparous women. Obstet Gynecol 1999;94:600–607.
7. Hincz P, Podciechowskil L, Grzesiak M, Horzelski W, Wilczyflski J. Epidural analgesia during labor:a retrospective cohort study on its effects on labour, delivery and neonatal outcome. Ginekol Pol 2014;85:923–928.
8. Lieberman E, O’donoghue C. Unintended effects of epidural analgesia during labor: a systematic review. Am J Obstet Gynecol 2002;186:S31–68.
9. Lim G, Facco FL, Nathan N, Waters JH, Wong CA, Eltzschig HK. A review of the impact of obstetric anesthesia on maternal and neonatal outcomes. Anesthesiology 2018;129:192–215.
10. Herrera-Gómez A, Luna-Bertos ED, Ramos-Torrecillas J, Ocaña-Peinado FM, Ruiz C, García-Martínez O. Risk assessments of epidural analgesia during labor and delivery. Clin Nurs Res 2018;27:841–852.
11. Kumar M, Chandra S, Ijaz Z, Senthilselvan A. Epidural analgesia in labour and neonatal respiratory distress: a case-control study. Arch Dis Child Fetal Neonatal Ed 2014;99:116–119.
12. Greenwell EA, Wyshak G, Ringer SA, Johnson LC, Rivkin MJ, Lieberman E. Intrapartum

temperature elevation, epidural use, and adverse outcome in term infants. Pediatrics 2012;129:e447–454.

13. Schrock SD, Harraway-Smith C. Labor analgesia. Am Fam Physician 2012;85:447–454.

14. Anim-Somuah M, Smyth RM, Cyna AM, Cuthbert A. Epidural versus non-epidural or no analgesia for pain management in labour. Cochrane Database Syst Rev 2018;5:CD000331.

15. Zhang J, Yancey MK, Klebanoff MA, Schwarz J, Schweitzer D. Does epidural analgesia prolong labor and increase risk of cesarean delivery? A natural experiment. Am J Obstet Gynecol 2001;185:128–134.

16. Naito Y, Ida M, Yamamoto R, Tachibana K, Kinouchi K. The effect of labor epidural analgesia on labor, delivery, and neonatal outcomes: a propensity score-matched analysis in a single Japanese institute. JA Clin Rep 2019;5:40.

17. Hu LQ, Zhang J, Wong CA, Cao Q, Zhang G, Rong H, et.al Impact of the introduction of neuraxial labor analgesia on mode of delivery at an urban maternity hospital in China. Int J Gynaecol Obstet 2015;129:17–21.

18. Pinheiro RL, Areia AL, Pinto AM, Donato H. Advanced maternal age: adverse outcomes of pregnancy, a meta-analysis. Acta Med Port 2019;32:219–226.

19. Dekker R, Breakey AA. Evidence on advanced maternal age. Evidence Based Birth 2016.

20. Wong CA. Epidural and spinal analgesia/Anesthesia for labor and vaginal delivery, Obstetric Anesthesia: Principles and Practice. In: Chestnut DH, editor. Anesthesiology. Mosby: Maryland Heights; 2014. pp 490.
21. Keriakos R, Sugumar S, Hilal N. Instrumental vaginal delivery--back to basics. J Obstet Gynaecol 2013;33:781–786.
22. Prior T, Kumar S. Mode of delivery has an independent impact on neonatal condition at birth. Eur J Obstet Gynecol Reprod Biol 2014;181:135–139.
23. Thavarajah H, Flatley C, Kumar S. The relationship between the five minute Apgar score, mode of birth and neonatal outcomes. J Matern Fetal Neonatal Med 2018;31:1335–1341.

Availability of Data and Materials:

The datasets analyzed during this study are available from the corresponding author on reasonable request.

Author Contributions:

All authors contributed to the study conception and design. Material preparation, data collection, and literature review were performed by SE. SE and SO performed data analysis. SE, YN, and SO wrote the original draft of the manuscript. MH supervised the study, including data analysis and interpretation. All authors commented on the previous version of the manuscript and approved the final manuscript.

Click here to access/download;Figure;Figure 1.tiff

Figure. 1

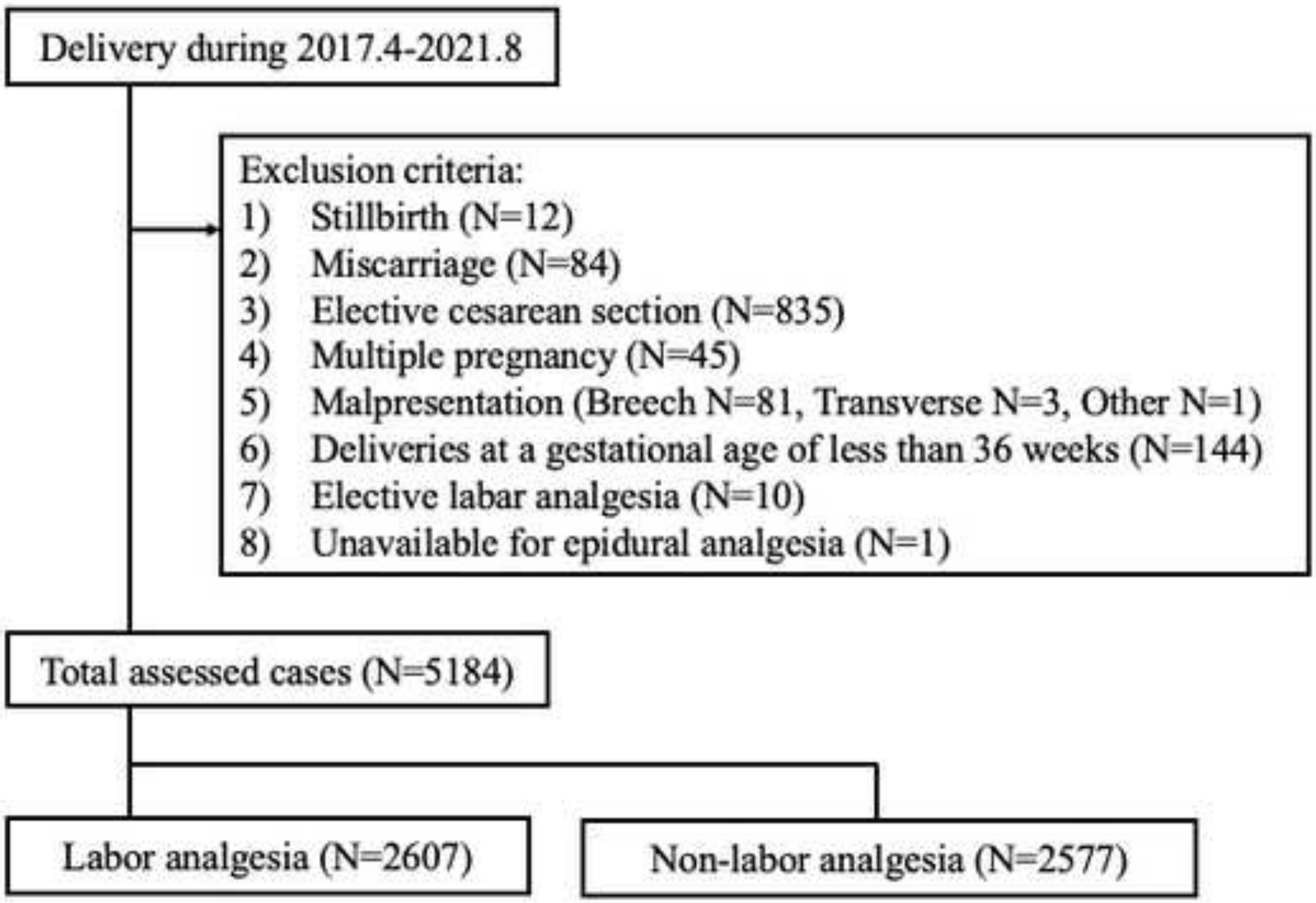

Click here to access/download
Supporting Information
STROBE checklist.docx

Full title: Impact of labor analgesia on mode of delivery and neonatal outcomes in Japan: A retrospective cohort study.

Short title: Impact of labor analgesia on delivery and neonates

Shu Eguchi[1*], Yuko Nagaoki[1], Sachiko Ohde[2], Michio Hirata, MD[1]

[1]Department of Pediatrics, St. Luke's International Hospital, Tokyo, Japan

[2]Graduate School of Public Health, St. Luke's International University, Tokyo, Japan

*Corresponding author

E-mail: ks0eh1nu0te17@gmail.com (SE)

Abstract

Labor analgesia (LA) is associated with the potential hazard of high-risk delivery, such as cesarean section (CS) and instrumental vaginal delivery (IVD), and adverse neonatal outcomes such as neonatal asphyxia and respiratory distress. Our objective was to examine the impact of LA on mode of delivery and neonatal outcomes and to counsel pregnant women about a potentially higher risk and allow them to decide LA or non-LA. This retrospective cohort study containing 5,184 pregnant women analyzed the association between LA and both mode of delivery and neonatal outcomes. LA increased the risk of IVD (Adjusted Odds Ratio [AOR] 3.25, 95% confidence interval [95%CI] 2.51–4.20) but decreased that of CS (AOR 0.52, 95%CI 0.44–0.60). Two factors (advanced maternal age [AOR 1.70, 95%CI 1.33–2.17] and primipara [AOR 4.72, 95%CI 3.30–6.75]) increased the risk of IVD. We should carefully consider the indication of LA for cases with these two factors since LA can increase the risk of IVD and adverse neonatal outcomes.

Introduction

Childbirth, as one of the most important life events for most women, requires them to go through a series of burdens during labor and delivery. The magnitude of labor pain is often compared with a finger amputation [1]. Although the pain itself is not life-threatening for pregnant women and infants, it can transiently impair the cognitive function of pregnant women, especially memory function, during early puerperium [2]. Furthermore, the pain is related to the risk of postpartum depression, and the degree of pain is also associated with the severity of depression [3]. Researchers reported that analgesia can relieve these symptoms [4]. Currently, labor analgesia (LA) with various methods is commonly used in modern obstetric practice and includes spinal anesthesia, epidural analgesia, and combined spinal-epidural analgesia [5]. However, the side effects, especially those related to the mode of delivery and neonatal outcomes, are of great concern. Some studies reported that LA is associated with the potential hazards of cesarean section (CS), instrumental vaginal delivery (IVD), and adverse neonatal outcomes [6–12]. While many studies from Europe and the United States concluded that epidural analgesia does not increase the risk of CS or adverse neonatal outcomes [13–15], Seybe et al. indicated that epidural analgesia in Asians may increase the risk of emergency CS (ECS) with an adjusted odds ratio (AOR) of 2.35 (95% confidence interval [95%CI] 1.04–5.34) [6].

The risk of ECS after application of epidural analgesia may vary depending on race, and thus, a race-specific analysis should be carefully considered. In Japan, where the rate of LA was traditionally significantly low at 6% according to the Japan Society of Obstetrics and Gynecology, compared with 20%–70% in other countries in Europe and the United States, the focus has been on the application of LA in the past 5 years. It is concerning that too few reports on LA in Japanese and Asian pregnant women have been published [16, 17]. Specifically, advanced maternal age is so remarkable in Japan that the maternal age at first birth rose from 25.7 years of age (1975) to 30.7 years of age (2020), according to the Ministry of Health, Labour and Welfare, Japan, and this trend could naturally increase the risk of ECS. A meta-analysis reported by Pinheiro et al. showed that advanced maternal age itself was associated with an increased risk of induced labor and CS [18]. Therefore, it is urgent that data from Japan be analyzed due to the high proportion of advanced maternal age.

We believe that this study, which includes an Asian population with a high rate of advanced maternal age, can contribute to the still controversial knowledge of the risks associated with LA. In our study, we used a retrospective chart review and multivariate analysis to examine the impact of labor analgesia on the mode of delivery and neonatal outcomes at our hospital and aimed to counsel pregnant women about a potentially higher risk and allow them

to decide LA or non-LA.

Materials and Methods

Study population

We conducted a retrospective cohort study of all pregnant women who were admitted for delivery from April 2017 to August 2021 at St Luke's International Hospital, Tokyo, Japan, where the average number of deliveries is 1,400 per annum. We selected samples of pregnant women based on the inclusion and exclusion criteria shown in Fig 1. We included all pregnant women who were admitted for delivery in the study periods. The exclusion criteria were as follows Stillbirths and miscarriages were excluded. Since a mode of delivery was defined as the primary outcome, cases with elective CS were excluded. For the same reason, cases with multiple pregnancy and malpresentation that would have resulted in elective CS were also excluded. Cases of delivery at a gestational age of less than 36 weeks were excluded because these could affect a mode of delivery and neonatal outcomes. Finally, cases with elective LA, cases in which LA was unavailable for LA, and cases with the lack of data due to outborn were excluded. All of the cases with elective LA had postoperative congenital heart disease, and the attending physician decided to conduct labor analgesia without the patient's wishes. The one patient with thrombocytopenia associated with Sjögren's syndrome who could not receive LA was excluded.

Fig 1. The study patient flow diagram.

Data collection

All data were obtained by chart review from the electronic medical records. Maternal data included age at delivery, which was dichotomized as advanced maternal age, defined as giving birth at age 35 or older [19], or not, height, pre-delivery body weight, pre-delivery body mass index, parity, duration of the second stage of labor, and whether or not LA was applied. Neonatal data included gestational age, gender, Apgar score (1 minute, 5 minute), admission to the neonatal intensive care unit (NICU), neonatal death, which is defined as death within 28 days of birth, and birth weight. The estimated fetal weight was replaced with the actual birth weight because the estimated fetal weight is not always accurate due to inter-examiner reliability. Pregnant women who received LA for vaginal delivery received either epidural analgesia, combined spinal-epidural analgesia, or dural puncture epidural.

Outcomes and definitions

The primary outcome of our study was the mode of delivery, and the secondary outcomes were neonatal admission to the NICU and Apgar score. The indications for admission to the NICU were gestational age less than 36 weeks, birth weight less than 2,300 g, the occurrence of a respiratory disorder requiring supplemental oxygen or more, and when deemed necessary by

neonatologists.

Statistical analysis

When the association between mode of delivery and application of LA was examined, we performed the analysis using two different outcomes related to the mode of delivery: spontaneous vaginal delivery (SVD) and ECS (“SVD versus ECS”) and SVD and IVD (“SVD versus IVD”) with the reasoning that the mode of delivery is often automatically determined by the degree of the fetal head station. When immediate delivery is considered, ECS was selected in cases of high fetal head station, while IVD was selected in cases of low fetal head station. The high fetal head station means at +2 or more. The final decision is made by the attending obstetrician. Multivariate logistic regression analysis was performed to calculate the AOR and 95%CI, to examine the impact of LA on the mode of delivery and neonatal outcomes. In addition, we focused only on pregnant women who received LA and attempted to identify the characteristics of patients with factors that make LA risky. This study was approved by the institutional review board of our institute (Approval code: 21-R113) and was conducted according to the principles of the Declaration of Helsinki. Since this study was an observational retrospective study, the requirement for written informed consent was waived, which allowed us to proceed with our study in an opt-out manner. All data were analyzed using SPSS Statistics

(IBM, Armonk, New York, USA). A result was considered statistically significant at $p < 0.05$.

Results

Patient characteristics

The total number of pregnant women who were admitted for delivery was 6,404. After considering inclusion and exclusion criteria, 5,184 women were finally included in the study population (Fig 1). The mean maternal age at delivery was 34.6 years with a standard deviation (SD) of 4.45 (Table 1).

Table 1. Patients characteristics / Labor analgesia group versus Non-labor analgesia group.

	All (N=5184)	Labor analgesia group (N=2607)	Non-labor analgesia group (N=2577)	p-value
Age (year-old)[a]	34.6 (4.45)	34.7 (4.42)	34.6 (4.49)	0.4191
Height (cm)[a]	160 (5.37)	160 (5.26)	160 (5.47)	1
Pre-delivery body weight (kg)[a]	52.4 (7.29)	52.4 (7.12)	52.5 (7.45)	0.6213
Pre-delivery BMI[a]	20.5 (2.62)	20.5 (2.56)	20.5 (2.67)	1
Primiparous[b]	3341 (64.4)	1892 (72.6)	1449 (56.2)	<0.001
Multiparous[b]	1843 (35.6)	715 (27.4)	1128 (43.8)	<0.001
Duration of second stage of labor[a]	12:33 (0:15)	15:38 (0:46)	9:04 (0:13)	<0.001
Mode of delivery[b]				
SVD	3750 (72.3)	1866 (71.6)	1884 (73.1)	0.218
IVD	412 (8.0)	331 (12.7)	81 (3.1)	<0.001
ECS	1022 (19.7)	410 (15.7)	612 (23.7)	<0.001
With labor analgesia[b]	2607 (50.3)	2607 (100)	0 (0.0)	0
Gestational age (weeks)[a]	39.5 (1.2)	39.6 (1.2)	39.4 (1.3)	<0.001
Male[b]	2594 (50.0)	1321 (50.7)	1273 (49.4)	0.359
Birth weight (g)[a]	3084 (377)	3120 (364)	3047 (387)	<0.001
Apgar score[a]				
1 minute	8.04 (0.936)	7.96 (1.05)	8.13 (0.797)	<0.001
5 minute	9.03 (0.603)	9.01 (0.628)	9.05 (0.576)	0.0169
Admission to NICU[b]	350 (6.8)	172 (6.6)	178 (6.9)	0.654
Neonatal death[b]	0 (0.0)	0 (0.0)	0 (0.0)	

BMI = Body mass index, ECS = Emergency cesarean section, IVD = Instrumental vaginal

delivery, NICU = Neonatal intensive care unit, SVD = Spontaneous vaginal delivery

[a]Mean (standard deviation), t-test p value.

[b]Number cases (percentage), chi-squared test p value.

The proportion of primipara was higher in the LA group (73%) than in the non-LA group (56%, $p < 0.001$) (Table 1). Primiparous women were more likely to receive LA (57%) than multiparous women (39%).

To examine the association between the mode of delivery and the application or not of LA, a univariate analysis was performed for IVD and ECS against SVD (Table 2). Compared with SVD, both IVD and ECS were associated with a higher proportion of admission to the NICU (IVD: 8.0%, $p = 0.008$ ECS: 12.8%, $p < 0.001$) and lower Apgar score (IVD 1 min/5 min: mean7.63/8.85, SD 1.09/0.603, $p < 0.001$/< 0.001 ECS 1 min/5 min: mean 7.56/8.82, SD 1.26/0.781, $p < 0.001$/< 0.001). The rate of LA was significantly higher in IVD (80%, $p < 0.001$) than SVD (50%) and conversely lower in ECS (40%, $p < 0.001$).

Table 2. Mode of delivery.

	SVD (N = 3750)	IVD (N = 412)	ECS (N = 1022)	p-value (SVD versus IVD)	p-value (SVD versus ECS)
Age (year-old)[a]	34.1 (4.36)	35.0 (4.54)	36.0 (4.47)	<0.001	<0.001
Height (cm)[a]	160 (5.27)	160 (5.22)	159 (5.67)	1	<0.001
Pre-delivery body weight (kg)[a]	52.2 (6.92)	51.9 (6.99)	53.5 (8.53)	0.4041	<0.001
Pre-delivery BMI[a]	20.4 (2.44)	20.3 (2.50)	21.2 (3.12)	0.4309	<0.001
Primiparous[b]	2112 (56.3)	361 (87.6)	868 (84.9)	<0.001	<0.001
Multiparous[b]	1638 (43.7)	51 (12.4)	154 (15.1)	<0.001	<0.001
Duration of second stage of labor[a]	11:33 (0:38)	16:12 (0:20)	17:04 (0:19)	<0.001	<0.001
With labor analgesia[b]	1866 (50.0)	331 (80.3)	410 (40.1)	<0.001	<0.001
Gestational age (weeks)[a]	39.4 (1.2)	39.6 (1.2)	39.5 (1.3)	0.0013	0.0204
Male[b]	1797 (47.9)	213 (51.7)	584 (57.1)	0.136	<0.001
Birth weight (g)[a]	3084 (355)	3084 (369)	3078 (451)	1	0.6525
Apgar score[a]					
1 minute	8.22 (0.733)	7.63 (1.09)	7.56 (1.26)	<0.001	<0.001
5 minute	9.11 (0.525)	8.85(0.603)	8.82 (0.781)	<0.001	<0.001
Admission to NICU[b]	186 (5.0)	33 (8.0)	131 (12.8)	0.008	<0.001
Neonatal death[b]	0 (0.0)	0 (0.0)	0 (0.0)		

BMI = Body mass index, ECS = Emergency cesarean section, IVD = Instrumental vaginal delivery, NICU = Neonatal intensive care unit, SVD = Spontaneous vaginal delivery

[a]Mean (standard deviation), t-test p value.

[b]Number cases (percentage), chi-squared test p value.

Primary outcome: Impact of labor analgesia on mode of delivery

First, we extracted the risks that may affect the mode of delivery and then examined the impact of LA on the mode of delivery. We hypothesized that the risks were LA, advanced maternal age, lower maternal height, larger estimated fetal weight, and primipara. The primary outcome for mode of delivery is shown in Table 3. We analyzed two groups: one compared SVD and ECS, excluding IVD (“SVD versus ECS”), and the other compared SVD and IVD, excluding ECS

("SVD versus IVD").

Table 3. The risk of instrumental vaginal delivery and emergency cesarean section, and adverse neonatal outcomes

	Adjusted Odds Ratios	95% Confidence interval	p-value
**ECS (versus SVD)**			
With labor analgesia	0.52	0.44 - 0.60	<0.001
Advanced maternal age (≥ 35 year-old)	2.39	2.05 - 2.78	<0.001
Height	0.95	0.94 - 0.97	<0.001
Estimated fetal weight	1.00	1.00 - 1.00	0.038
Primiparous	6.00	4.95 - 7.28	<0.001
**IVD (versus SVD)**			
With labor analgesia	3.25	2.51 - 4.20	<0.001
Advanced maternal age (≥ 35 year-old)	1.66	1.33 - 2.05	<0.001
Height	0.99	0.97 - 1.01	0.386
Estimated fetal weight	1.00	1.00 - 1.00	0.741
Primiparous	5.13	3.77 - 6.99	<0.001
**IVD (versus SVD) among patients with labor analgesia**			
Advanced maternal age (≥ 35 year-old)	1.70	1.33 - 2.17	<0.001
Height	0.98	0.96 - 1.01	0.167
Estimated fetal weight	1.00	1.00 - 1.00	0.643
Primiparous	4.72	3.30 - 6.75	<0.001
**IVD (versus SVD) among patients without labor analgesia**			
Advanced maternal age (≥ 35 year-old)	1.49	0.94 - 2.35	0.093
Height	1.02	0.97 - 1.06	0.481
Estimated fetal weight	1.00	1.00 - 1.00	0.895
Primiparous	6.23	3.37 - 11.52	<0.001
**Apgar score 1-minute <7**			
With labor analgesia	2.06	1.50 - 2.83	<0.001
Advanced maternal age (≥ 35 year-old)	1.29	0.96 - 1.74	0.088
Height	0.99	0.96 - 1.02	0.379
Estimated fetal weight	1.00	1.00 - 1.00	0.452
Primiparous	2.68	1.80 - 3.9	<0.001
**Apgar score 5-minute <7**			
With labor analgesia	1.13	0.56 - 2.27	0.741
Advanced maternal age (≥ 35 year-old)	1.53	0.76 - 3.09	0.234
Height	0.92	0.86 - 0.99	0.015
Estimated fetal weight	1.00	1.00 - 1.00	0.227
Primiparous	2.07	0.88 - 4.87	0.096
**Admission to NICU**			
With labor analgesia	1.06	0.85 - 1.33	0.615
Advanced maternal age (≥ 35 year-old)	1.34	1.07 - 1.68	0.011
Height	1.01	0.99 - 1.03	0.338
Estimated fetal weight	1.00	1.00 - 1.00	<0.001
Primiparous	1.68	1.30 - 2.18	<0.001

ECS = Emergency cesarean section, IVD = Instrumental vaginal delivery, NICU = Neonatal intensive care unit, SVD = Spontaneous vaginal delivery

In "SVD versus ECS," those of advanced maternal age and primipara exhibited a higher AOR (AOR 2.39, 6.00 95%CI 2.05–2.78, 4.95–7.28). Lower maternal height and larger estimated fetal weight increased the risks of ECS, while the use of LA decreased the risk of ECS (AOR 0.52, 95%CI 0.44–0.60).

Similar to the "SVD versus ECS" results, advanced maternal age and primipara also exhibited a higher AOR (AOR 1.66, 5.13 95%CI 1.33–2.05, 3.77–6.99) in the "SVD versus IVD" comparison. Maternal height and estimated fetal weight were not associated with the increased risk of IVD, but the use of LA increased the risk of IVD (AOR 3.25, 95%CI 2.51–4.20).

Secondary outcome: Impact of labor analgesia on neonatal outcomes

The secondary outcomes, including neonatal outcomes and Apgar score, are shown in Table 3. LA lowered the Apgar score at 1 minute (AOR 2.06, 95%CI 1.50–2.83), but it did not influence the Apgar score at 5 minutes or the NICU admission rate. Being a primipara was the strongest

factor that could cause all adverse neonatal outcomes after adjusting for covariates including LA prescription.

When the population was restricted to only those who received LA, we found that pregnant women with the following two risk factors demonstrated a higher risk of IVD: advanced maternal age (AOR 1.70, 95%CI 1.33–2.17) and primipara (AOR 4.72, 95%CI 3.30–6.75) (Table 3). Neither lower maternal height nor larger estimated fetal weight were significantly associated with an increased risk of IVD .

Discussion

This study included more than 5,000 pregnant women, approximately half of whom were of advanced maternal age, and who were admitted to our hospital for delivery from 2017 to 2021. In a multivariate analysis, LA increased the risk of IVD but decreased that of ECS. Moreover, advanced maternal age and primipara increased the risks of both ECS and IVD. Among those who received LA, two factors of advanced maternal age and primipara increased the risk of IVD; the AOR of primipara was highest at 4,72 (95%CI 3.30–6.75) and that of advanced maternal age was 1.70 (95%CI 1.33–2.17).

To the best of our knowledge, our study is one of few reports from Japan that presents an association between LA and mode of delivery as well as neonatal outcomes. The novelty is that we sought to identify the characteristics of pregnant women who may need to be cautious in selecting LA. Our study is valuable in that we analyzed primarily Asian patients, who are recognized to be at an increased risk of CS [6]; we also included a large number of patients of advanced maternal age.

As our results showed that LA decreased the risk of CS, many recent reports showed that LA is not associated with increased risk of CS or that LA even reduces CS rates [13, 15]. Despite a previous study published several decades ago, which concluded that epidural

analgesia was associated with a significantly increased risk of CS among primipara [6], a recent Cochrane Review in 2018 that included 40 trials and more than 11,000 pregnant women found that epidural analgesia did not increase the risk of CS [14]. The reason that LA is not associated with and does not increase the ECS rate may be because factors associated with more painful labor are themselves associated with an increased risk of CS (e.g., fetal malrotation, fetal-pelvic disproportion, dysfunctional labor) [20]. Thus, we consider that pain relief might help to avoid ECS. However, no studies compared the degree of pain with the CS rate, and it is unclear why LA lowers the CS rate. Further investigation is needed to determine the pathophysiological reasons.

Our results showed that LA also increases the risk of IVD. While some studies reported that LA is not associated with the increased risk of IVD [15], many other studies, including a systematic review by Lieberman et al. [8], reported the association between LA and an increased risk of IVD [7, 9, 10, 13]. Lieberman et al. estimated a pooled odds ratio of 2.2 (95%CI 1.3–7.8) [8], which is comparable to the result of our study (AOR 3.25, 95%CI 2.51–4.20), and considered that the most common reason why pregnant women who receive LA are likely to undergo IVD is due to stalled labor, followed by risk to fetal well-being [8].

We found that the use of LA is associated with adverse neonatal outcomes. The effects

of LA on neonatal outcomes are controversial and are the same as those on mode of delivery. Although a meta-analysis reported in the Cochrane Review in 2018 denied an association between epidural analgesia and adverse neonatal outcomes, such as admission rate to the NICU and an Apgar score of less than 7 at 5 minutes [14], many other studies confirmed the association [7, 11, 12]. In general, Apgar scores are lower in newborns delivered by CS [22, 23], but in our study, the Apgar score was lower in the LA group even though the CS rate decreased, which suggests that LA itself must lower the Apgar score. Kumar et al. reported that the Apgar score was significantly lower with use of epidural analgesia because of respiratory distress, and they considered that a large volume of distribution at a steady state in neonates resulted in a much longer terminal elimination half-life of fentanyl over several hours [11]. Most newborns with neonatal asphyxia are able to establish fetal-to-neonatal transition after appropriate resuscitation immediately after birth. This means that even if the Apgar score at 1 minute is low, the Apgar score at 5 minutes will be higher after appropriate resuscitation. This may be the reason why the results of our study did not reveal statistically significant differences when the Apgar score at 5 minutes was less than 7. Researchers suggested that LA may elicit more severe adverse effects in a newborn in the immediate postnatal period than what the Apgar score at 1 minute purportedly represents. Neonatologists are expected to exhibit resuscitation

skills for cases of neonatal asphyxia, especially in cases of LA. To promote resuscitation skills, we should provide a training and educational system to maintain higher skills and knowledge. Even if neonatal resuscitation is learned through simulation, this skill is difficult to maintain without practical experience, and thus, aggressive resuscitation practices are important. The centralization of facilities where LA is available might ensure safe deliveries. We should carefully consider LA, especially in facilities without personnel, such as pediatricians, neonatologists, and midwives, who attend deliveries.

According to our results, LA may reduce the risk of ECS but increase the risk of IVD and lower the Apgar score at 1 minute. In particular, the increased risk of IVD was significantly higher when the pregnant women who were primiparous and of advanced maternal age were administered LA. While the decrease in ECS is favorable in terms of surgical invasiveness and the safety of the next delivery, the increase in IVD is a serious issue. IVD is associated with many potential risks to both the mother (e.g., perineal and vaginal tears) and the newborn (e.g., brachial plexus injury with shoulder dystocia, subgaleal and intracranial hemorrhage) [21]. Prior et al. reported that the Apgar score was lower in infants delivered by IVD than in those delivered by CS [22]. Based on our results, we suggest that pregnant women at an increased risk of IVD, such as those of advanced maternal age (i.e., 35 years of age or older), lower

maternal height, larger estimated fetal weight, and primipara, should carefully consider LA. Hence, when obstetricians and/or anesthesiologists confirm a pregnant woman's requests to receive LA, explaining not only the advantages but also the disadvantages and risks and obtaining appropriate informed consent is important. Moreover, pregnant women with these risk factors may be guided to receive alternative treatments, such as acupuncture, hypnotism, yoga, exercise during pregnancy, hydrotherapy, transcutaneous electronic nerve stimulation, massage, and relaxation techniques [5]. Among IVD techniques, obstetric forceps are particularly dangerous in the hands of inexperienced obstetricians. Therefore, LA should be administered only in facilities with well-trained and experienced obstetricians because pregnant women who receive LA are more likely to require IVD.

The results of our study are based on a limited population, and as mentioned above, we should be cautious about perceptions of the indications for and the risks of LA. Determining the indications for LA on a case-by-case basis and recognizing that LA is not beneficial for every pregnant woman is necessary because the population of pregnant women and facilities vary depending on the country, region, and other circumstances. The environment surrounding childbirth is changing; for example, advanced maternal age is increasing in all developed countries due to women's career advancement and lifestyle changes, and thus, we need to

consistently update our research findings on the impacts of LA. In addition, the medical principle behind lower Apgar scores is not completely understood, and since the topic of LA remains controversial, further research is needed.

Our study also exhibits some limitations. The impact of the method of LA on the mode of delivery was not considered. In Tables 1 and 2, although statistically significant differences were observed in terms of maternal age, height, pre-delivery body weight, pre-delivery body mass index, gestational age, sex of newborn, and birth weight, these differences are not noteworthy in clinical practice because these factors were continuous variables and the number of cases that met the inclusion criteria was high. Similarly, in the multivariate analysis, we should focus on the interpretation of the comparison of the AOR of continuous variables, such as maternal height and estimated birth weight, and that of categorical variables, such as LA, advanced maternal age, and primipara. In addition, establishing the cut-off for maternal height and estimated birth weight is very difficult, and thus, the results of our study are unlikely to affect actual practice without specific values.

In conclusion, although LA is an important means to relieve pain during labor and delivery, it can increase the risk of IVD and an Apgar score of less than 7 at 1 minute. Therefore, we should carefully consider the indication of LA in cases of advanced maternal age and

primipara. Furthermore, it is desirable to maintain and improve resuscitation skills through training and certification systems and to intensify facilities where LA is available.

Acknowledgements

Not applicable.

References

1. Melzack R. The myth of painless childbirth (the John J. Bonica lecture). Pain 1984;19:321–337.
2. Eidelman AI, Hoffmann NW, Kaitz M. Cognitive deficits in women after child-birth. Obstet Gynecol 1993;81:764–767.
3. Eisenach JC, Pan PH, Smiley R, Lavand'homme P, Landau R, Houle TT. Severity of acute pain after childbirth, but not type of delivery, predicts persistent pain and postpartum depression. Pain 2008;140(1):87–94.
4. Hawkins JL. Epidural analgesia for labor and delivery. N Engl J Med 2010;362:1503–1510.
5. Koyyalamudi V, Sidhu G, Cornett EM, Nguyen V, Labrie-Brown C, Fox CJ, et al. New labor pain treatment options. Curr Pain Headache Rep 2016;20:11.

6. Seyb ST, Berka RJ, Socol ML, Dooley SL. Risk of cesarean delivery with elective induction of labor at term in nulliparous women. Obstet Gynecol 1999;94:600–607.
7. Hincz P, Podciechowskil L, Grzesiak M, Horzelski W, Wilczyflski J. Epidural analgesia during labor:a retrospective cohort study on its effects on labour, delivery and neonatal outcome. Ginekol Pol 2014;85:923–928.
8. Lieberman E, O'donoghue C. Unintended effects of epidural analgesia during labor: a systematic review. Am J Obstet Gynecol 2002;186:S31–68.
9. Lim G, Facco FL, Nathan N, Waters JH, Wong CA, Eltzschig HK. A review of the impact of obstetric anesthesia on maternal and neonatal outcomes. Anesthesiology 2018;129:192–215.
10. Herrera-Gómez A, Luna-Bertos ED, Ramos-Torrecillas J, Ocaña-Peinado FM, Ruiz C, García-Martínez O. Risk assessments of epidural analgesia during labor and delivery. Clin Nurs Res 2018;27:841–852.
11. Kumar M, Chandra S, Ijaz Z, Senthilselvan A. Epidural analgesia in labour and neonatal respiratory distress: a case-control study. Arch Dis Child Fetal Neonatal Ed 2014;99:116–119.
12. Greenwell EA, Wyshak G, Ringer SA, Johnson LC, Rivkin MJ, Lieberman E. Intrapartum

temperature elevation, epidural use, and adverse outcome in term infants. Pediatrics 2012;129:e447–454.

13. Schrock SD, Harraway-Smith C. Labor analgesia. Am Fam Physician 2012;85:447–454.

14. Anim-Somuah M, Smyth RM, Cyna AM, Cuthbert A. Epidural versus non-epidural or no analgesia for pain management in labour. Cochrane Database Syst Rev 2018;5:CD000331.

15. Zhang J, Yancey MK, Klebanoff MA, Schwarz J, Schweitzer D. Does epidural analgesia prolong labor and increase risk of cesarean delivery? A natural experiment. Am J Obstet Gynecol 2001;185:128–134.

16. Naito Y, Ida M, Yamamoto R, Tachibana K, Kinouchi K. The effect of labor epidural analgesia on labor, delivery, and neonatal outcomes: a propensity score-matched analysis in a single Japanese institute. JA Clin Rep 2019;5:40.

17. Hu LQ, Zhang J, Wong CA, Cao Q, Zhang G, Rong H, et.al Impact of the introduction of neuraxial labor analgesia on mode of delivery at an urban maternity hospital in China. Int J Gynaecol Obstet 2015;129:17–21.

18. Pinheiro RL, Areia AL, Pinto AM, Donato H. Advanced maternal age: adverse outcomes of pregnancy, a meta-analysis. Acta Med Port 2019;32:219–226.

19. Dekker R, Breakey AA. Evidence on advanced maternal age. Evidence Based Birth 2016.

20. Wong CA. Epidural and spinal analgesia/Anesthesia for labor and vaginal delivery, Obstetric Anesthesia: Principles and Practice. In: Chestnut DH, editor. Anesthesiology. Mosby: Maryland Heights; 2014. pp 490.

21. Keriakos R, Sugumar S, Hilal N. Instrumental vaginal delivery--back to basics. J Obstet Gynaecol 2013;33:781–786.

22. Prior T, Kumar S. Mode of delivery has an independent impact on neonatal condition at birth. Eur J Obstet Gynecol Reprod Biol 2014;181:135–139.

23. Thavarajah H, Flatley C, Kumar S. The relationship between the five minute Apgar score, mode of birth and neonatal outcomes. J Matern Fetal Neonatal Med 2018;31:1335–1341.

Availability of Data and Materials:

The datasets analyzed during this study are available from the corresponding author on reasonable request.

Author Contributions:

All authors contributed to the study conception and design. Material preparation, data collection, and literature review were performed by SE. SE and SO performed data analysis. SE, YN, and SO wrote the original draft of the manuscript. MH supervised the study, including data analysis and interpretation. All authors commented on the previous version of the manuscript and approved the final manuscript.

Dear Emily Chenette, Editor-in-Chief of *PLOS ONE*

On behalf of all co-authors of this manuscript, I would like to thank the editor and the reviewers for a thorough review of our manuscript and constructive advice, which have improved the manuscript considerably. We carefully went over the important comments/suggestions from the reviewers and revised the manuscript. Please find our point-by-point responses to the reviewers' comments below. All changes we made in this revision are highlighted in yellow. We hope that you will find the revisions and comments satisfactory.

Reviewer #1:

#1. Your institute often accept the foreign pregnant women from many countries. Is it necessary to discuss about the racial difference in this study?

Response to the Reviewer:

We appreciate the reviewer's question. We think that it was difficult to strictly extract race including one half or one quarter patients and exclude them. It is true that our hospital sees many foreign patients, but the majority of pregnant women who deliver in our hospital is Japanese. Furthermore, the majority of the few foreign patients is Chinese or Korean, thus we believe that not excluding the foreign patients does not have a significant impact on the results of this study dealing with Asian.

#2. I would like to know that the precise indications for deciding IVD, which may help to clear the precise delivery outcome on LA.

Response to the Reviewer:

In our hospital, when immediate delivery is considered, cesarean section is selected in cases of high fetal head station, while instrumental vaginal delivery is selected in cases of low fetal head station. The high fetal head station means at +2 or more. The final decision is made by the attending obstetrician. I have added the above text to the main text. (Page 8, Line 103–104)

#3. In Table 3, only the AOR and 95 % CI have shown in the IVD vs SVD with LA patients and suggested significant differences. However, the differences in estimated body weight among three groups (SVD, IVD and ECS) seem much closer and suggest no significant differences in Table 1. To avoid the misrecognition you should suggest more precise data in Table 3 or in the draft.

Response to the Reviewer:

In Table 1, the univariate analysis was performed, whereas in Table 3, the multivariate analysis was performed. Not only the method of analysis but also the number of cases covered was different. The body weight was a continuous variable, whereas the sample size was large, which is the reason for the significant difference, and we consider that the significant difference has a little clinical significance.

Reviewer #2:

Although your manuscript is interesting and well-written, I think it does not have merit enough for publication in PLOS One, because it is only a retrospective cohort (please, include the study design in the title). There are lots of randomized controlled trials demonstrating maternal and neonatal outcomes with labor analgesia and a Cochrane systematic review with more than 11,000 patients included. I suggest you try another journal and follow STROBE recommendations for observational studies, with a copy of STROBE checklist for cohort studies.

Response to the Reviewer:

We appreciate the reviewer’s peer review. We followed the reviewer's advice to add the study design to the title and submit the STROBE checklist. Although, several retrospective cohort studies have been published before, there are very few reports from Asia. We consider that the present study has great clinical significance with increase of the advanced maternal age and popularization of the labor analgesia.

Reviewer #3:

Abstract:

1. What adverse neonatal outcomes are you specifically considering? It is mentioned that labor analgesia increases adverse neonatal outcomes, so perhaps mentions one or two examples.

Response to the Reviewer:

We appreciate the reviewer's advice. The adverse neonatal outcomes mean such as neonatal asphyxia and respiratory distress. I have added the examples to the main text. (Page 2, Line 16)

2. The idea that the premise of this study was to identify people who should avoid labor analgesia seems problematic from the start. Perhaps the focus should be on counseling patients about a potentially higher risk of needing an operative vaginal delivery and allowing the patient to decide rather than advising them that they can't get analgesia.

Response to the Reviewer:

As the reviewer's advice, the aim is to counsel patients and allow them to make informed decision. We followed the advice and revised. (Page 2, Line 17–18)

Introduction:

3. May be worth mentioning that labor pain is also associated with postpartum depression and anxiety.

Response to the Reviewer:

We followed the advice and revised with the additional reference. (Page 3, Line 31–33)

4. I'm again troubled that the focus of this analysis is to identify people who should avoid labor analgesia. There are outcomes worse than requiting an operative delivery, and pain and depression may be among such outcomes.

Response to the Reviewer:

As we responded in the question 2, we revised the statement to focus on counseling and allowing patients to decide rather than advising them that they can't receive labor analgesia. (Page 4, Line 59–Page 5, Line 60)

Methods:

5. "The estimated fetal weight was substituted for the actual birth weight because the estimated fetal weight is not always accurate due to inter-examiner reliability." Consider

re-wording this as “The estimated fetal weight was replaced with the actual birth weight…”

Response to the Reviewer:

We followed the advice and revised. (Page 7, Line 86)

6. Consider rewording this “When termination is considered,” as “When immediate delivery is considered” since termination has many meanings.

Response to the Reviewer:

We followed the advice and revised. (Page 8, Line 101)

7. What are the indications for operative delivery at this institution? This may inform concerns related to this rate being higher. Many countries would interpret an increase in operative delivery over cesarean to be a success.

Response to the Reviewer:

In our hospital, when immediate delivery is considered, cesarean section is selected in cases of high fetal head station, while instrumental vaginal delivery is selected in cases of low fetal head station. The high fetal head station means at +2 or more. The final decision is made by the attending obstetrician. I have added the above text to the main text. (Page 8, Line 103–104)

As the reviewer concerns, a decrease of cesarean sections is desirable in obstetric aspects. In this study, we would like to argue that labor analgesia brings not only good things but also bad things in neonatal aspect. Therefore, following the reviewer’s advice, we revised the statement to focus on counseling and allowing patients to decide rather than advising them that they can't receive labor analgesia. (Page 4, Line 59–Page 5, Line 60)

Results:

8. “When the population was restricted to only those who received LA, we found that pregnant women with the following four risk factors demonstrated a higher risk of IVD: advanced maternal age, lower maternal height, larger estimated fetal weight, and primipara (Table 3).” To be fair, you should do the same analysis on those who did NOT receive analgesia because you might find the same risk factors for operative delivery, so the analgesia may not have impacted this as much as you propose.

9. Indeed, for advanced maternal age and some other factors, analyzing only those with

analgesia has a lower aOR than when you included everyone. Please also do the same analysis on those without analgesia.

Response to the Reviewer:

As suggested, we performed the same analysis as follows. In addition, some modification was required in the analysis data.

IVD (versus SVD) everyone			
Advanced maternal age (≥ 35 year-old)	1.76	1.43 - 2.18	<0.001
Height	0.99	0.97 - 1.01	0.167
Estimated fetal weight	1.00	1.00 - 1.00	0.191
Primiparous	6.34	4.67 - 8.61	<0.001
**IVD (versus SVD) among patients with labor analgesia**			
Advanced maternal age (≥ 35 year-old)	1.70	1.33 - 2.17	<0.001
Height	0.98	0.96 - 1.01	0.167
Estimated fetal weight	1.00	1.00 - 1.00	0.643
Primiparous	4.72	3.30 - 6.75	<0.001
**IVD (versus SVD) among patients without labor analgesia**			
Advanced maternal age (≥ 35 year-old)	1.49	0.94 - 2.35	0.093
Height	1.02	0.97 - 1.06	0.481
Estimated fetal weight	1.00	1.00 - 1.00	0.895
Primiparous	6.23	3.37 - 11.52	<0.001

Among patients without labor analgesia, only primiparous had higher AOR, whereas, among those with labor analgesia, not only primiparous but also advanced maternal age are associated with increased risk of IVD. The results of the analysis are provided in this letter and some have been added to Table 3 in the revised manuscript.

10. The discussion mentions that operative delivery is associated with perineal tearing but this was not actually evaluated in this study. Please consider adding this analysis as it would help advise the results and discussion.

Response to the Reviewer:

Certainly, perineal tearing is one of the important complications of instrumental vaginal delivery. However, the outcome of the present study are the mode of delivery and the neonatal outcomes, and the complications that occur after delivery was not our interest. In fact that the perineal tearing is highly recognized as a complication of instrumental vaginal delivery is referred in the manuscript [20].

Discussion:

11. The aforementioned additional analysis on those who did NOT receive analgesia must be performed. These results should be reviewed after this sentence: “Among those who

received LA, all four factors of advanced maternal age, lower maternal height, larger estimated fetal weight, and primipara increased the risk of IVD; the AOR of primipara was highest at 6.76 (95%CI 5.10–8.96) and that of advanced maternal age was second highest at 1.72 (95%CI 1.43–2.06).”

Response to the Reviewer:

Please refer the response to Comment 8 and 9 above.

12. I remain unconvinced of the conclusions but await additional analyses.

Response to the Reviewer:

We have made corrections as described above. We appreciate your re-review.

Reviewer #4:

In the Abstract

Adjusted Odds Ratio - put the confidence interval in the results. Introduction Last paragraph - just put the purpose of the study. It is not the place to describe the methodology.

Response to the Reviewer:

We followed the advice and revised. (Page 2, Line 21–23)

In Materials and Methods

Describe inclusion and exclusion criteria. Figure 1 contains the exclusion criteria and does not have the inclusion ones. Both should be described in the methodology.

Response to the Reviewer:

We followed the advice and revised. (Page 6, Line 66–77)

Lines 62 to 65 - the included text is disconnected from the rest.

Response to the Reviewer:

We confirmed that the submission guideline stated, "Figure captions must be inserted in the text of the manuscript, immediately following the paragraph in which the figure is first cited (read order).". We would appreciate if the reviewer could point out specifically if we seem to be mistaken.

Bibliographic references

Standardize the presentation of bibliographic references.

Reference 19 - has the year of publication in 2 locations.

As can be seen below: 19.Wong CA. (2014). Epidural and spinal analgesia/Anesthesia for labor and vaginal delivery, Obstetric Anesthesia: Principles and Practice. In: Chestnut DH (ed). Anesthesiology. Mosby: Maryland Heights, 2014, pp 490.

Response to the Reviewer:

We followed the advice and revised. (Page 25, Line 331–333)

Reviewer #5:

In general, the manuscript is fit for publication. In discussion section, data should be analyzed carefully, and conclusion should be made cautiously. Adverse neonatal and fetal outcome, cesarean section and instrumental vaginal delivery may be strongly associated with and even may be caused by advanced maternal age, lower maternal height, larger fetal weight, and primipara, and other risk factors, but not by LA. Perhaps, LA should not be restricted or prohibited in these pregnant women and those in labor with the four kinds of risk factors mentioned above. This should be re-analyzed carefully and added into the discussion section.

Response to the Reviewer:

We appreciate the reviewer's advice. We did the same analysis on those without labor analgesia. The results of the analysis are provided in this letter and have been added to Table 3 in the revised manuscript.

IVD (versus SVD) everyone			
Advanced maternal age (≥ 35 year-old)	1.76	1.43 - 2.18	<0.001
Height	0.99	0.97 - 1.01	0.167
Estimated fetal weight	1.00	1.00 - 1.00	0.191
Primiparous	6.34	4.67 - 8.61	<0.001
**IVD (versus SVD) among patients with labor analgesia**			
Advanced maternal age (≥ 35 year-old)	1.70	1.33 - 2.17	<0.001
Height	0.98	0.96 - 1.01	0.167
Estimated fetal weight	1.00	1.00 - 1.00	0.643
Primiparous	4.72	3.30 - 6.75	<0.001
**IVD (versus SVD) among patients without labor analgesia**			
Advanced maternal age (≥ 35 year-old)	1.49	0.94 - 2.35	0.093
Height	1.02	0.97 - 1.06	0.481
Estimated fetal weight	1.00	1.00 - 1.00	0.895
Primiparous	6.23	3.37 - 11.52	<0.001

As the reviewer's advice, the aim is to counsel patients and allow them to make informed decision. We followed the advice and revised. (Page 2, Line 17–18, Page 4, Line 59–Page 5, Line 60)

Reviewer #6:

Page 5 line 63 and 64 : sentence are not clear. It is better to write the criteria inclusion in narrative.

Response to the Reviewer:

We followed the advice and revised.

Page 8 line 105 = table title not complete should be completed with place, and when?, n=??

Page 9 line = 110-111 should be deleted, should not be written here.

Page 9 line 125 : table title not complete should be completed with place, and when?, n=??

Page 10 line 127-128 should be deleted, should not be written here.

Page11 line 137-138 table title not complete should be completed with place, and when?, n=??

Page 11 line 140 and page 12 line 141 should be deleted, should not be written here

Response to the Reviewer:

We appreciate the reviewer's peer review. We adhere to the guidelines when submitting.

We humbly request your further advice.

Page 11 line 149 sentence is not clear

Response to the Reviewer:

We followed the advice and revised. (Page 14, Line 163–164)

Page 17 Line 217-222; sentence and statement is not clear

Response to the Reviewer:

We followed the advice and revised. (Page 19, Line 229–230)

Sincerely yours,

Shu Eguchi
Department of Pediatrics
St. Luke’s International Hospital
