# Supplementary material for: Impact of labor analgesia on mode of delivery and neonatal outcomes in Japan: A retrospective cohort study
Source: PLoS One. 2023 Apr 12;18(4):e0284368. doi: 10.1371/journal.pone.0284368 (PMC10096281; doi:10.1371/journal.pone.0284368)
Supplement: Attachment — Submitted filename: Response to Reviewers.docx [file pone.0284368.s003.docx]

Dear Emily Chenette, Editor-in-Chief of *PLOS ONE*

On behalf of all co-authors of this manuscript, I would like to thank the editor and the reviewers for a thorough review of our manuscript and constructive advice, which have improved the manuscript considerably. We carefully went over the important comments/suggestions from the reviewers and revised the manuscript. Please find our point-by-point responses to the reviewers’ comments below. All changes we made in this revision are highlighted in yellow. We hope that you will find the revisions and comments satisfactory.

Reviewer #1:

#1. Your institute often accept the foreign pregnant women from many countries. Is it necessary to discuss about the racial difference in this study?

Response to the Reviewer:

We appreciate the reviewer’s question. We think that it was difficult to strictly extract race including one half or one quarter patients and exclude them. It is true that our hospital sees many foreign patients, but the majority of pregnant women who deliver in our hospital is Japanese. Furthermore, the majority of the few foreign patients is Chinese or Korean, thus we believe that not excluding the foreign patients does not have a significant impact on the results of this study dealing with Asian.

#2. I would like to know that the precise indications for deciding IVD, which may help to clear the precise delivery outcome on LA.

Response to the Reviewer:

In our hospital, when immediate delivery is considered, cesarean section is selected in cases of high fetal head station, while instrumental vaginal delivery is selected in cases of low fetal head station. The high fetal head station means at +2 or more. The final decision is made by the attending obstetrician. I have added the above text to the main text. (Page 8, Line 103–104)

#3. In Table 3, only the AOR and 95 % CI have shown in the IVD vs SVD with LA patients and suggested significant differences. However, the differences in estimated body weight among three groups (SVD, IVD and ECS) seem much closer and suggest no significant differences in Table 1. To avoid the misrecognition you should suggest more precise data in Table 3 or in the draft.

Response to the Reviewer:

In Table 1, the univariate analysis was performed, whereas in Table 3, the multivariate analysis was performed. Not only the method of analysis but also the number of cases covered was different. The body weight was a continuous variable, whereas the sample size was large, which is the reason for the significant difference, and we consider that the significant difference has a little clinical significance.

Reviewer #2:

Although your manuscript is interesting and well-written, I think it does not have merit enough for publication in PLOS One, because it is only a retrospective cohort (please, include the study design in the title). There are lots of randomized controlled trials demonstrating maternal and neonatal outcomes with labor analgesia and a Cochrane systematic review with more than 11,000 patients included. I suggest you try another journal and follow STROBE recommendations for observational studies, with a copy of STROBE checklist for cohort studies.

Response to the Reviewer:

We appreciate the reviewer’s peer review. We followed the reviewer's advice to add the study design to the title and submit the STROBE checklist. Although, several retrospective cohort studies have been published before, there are very few reports from Asia. We consider that the present study has great clinical significance with increase of the advanced maternal age and popularization of the labor analgesia.

Reviewer #3:

Abstract:

1. What adverse neonatal outcomes are you specifically considering? It is mentioned that labor analgesia increases adverse neonatal outcomes, so perhaps mentions one or two examples.
Response to the Reviewer:

We appreciate the reviewer’s advice. The adverse neonatal outcomes mean such as neonatal asphyxia and respiratory distress. I have added the examples to the main text. (Page 2, Line 16)

2. The idea that the premise of this study was to identify people who should avoid labor analgesia seems problematic from the start. Perhaps the focus should be on counseling patients about a potentially higher risk of needing an operative vaginal delivery and allowing the patient to decide rather than advising them that they can’t get analgesia.

Response to the Reviewer:

As the reviewer’s advice, the aim is to counsel patients and allow them to make informed decision. We followed the advice and revised. (Page 2, Line 17–18)

Introduction:

3. May be worth mentioning that labor pain is also associated with postpartum depression and anxiety.

Response to the Reviewer:

We followed the advice and revised with the additional reference. (Page 3, Line 31–33)

4. I’m again troubled that the focus of this analysis is to identify people who should avoid labor analgesia. There are outcomes worse than requiting an operative delivery, and pain and depression may be among such outcomes.

Response to the Reviewer:

As we responded in the question 2, we revised the statement to focus on counseling and allowing patients to decide rather than advising them that they can’t receive labor analgesia. (Page 4, Line 59–Page 5, Line 60)

Methods:

5. “The estimated fetal weight was substituted for the actual birth weight because the estimated fetal weight is not always accurate due to inter-examiner reliability.” Consider re-wording this as “The estimated fetal weight was replaced with the actual birth weight…”

Response to the Reviewer:

We followed the advice and revised. (Page 7, Line 86)

6. Consider rewording this “When termination is considered,” as “When immediate delivery is considered” since termination has many meanings.

Response to the Reviewer:

We followed the advice and revised. (Page 8, Line 101)

7. What are the indications for operative delivery at this institution? This may inform concerns related to this rate being higher. Many countries would interpret an increase in operative delivery over cesarean to be a success.

Response to the Reviewer:

In our hospital, when immediate delivery is considered, cesarean section is selected in cases of high fetal head station, while instrumental vaginal delivery is selected in cases of low fetal head station. The high fetal head station means at +2 or more. The final decision is made by the attending obstetrician. I have added the above text to the main text. (Page 8, Line 103–104)

As the reviewer concerns, a decrease of cesarean sections is desirable in obstetric aspects. In this study, we would like to argue that labor analgesia brings not only good things but also bad things in neonatal aspect. Therefore, following the reviewer’s advice, we revised the statement to focus on counseling and allowing patients to decide rather than advising them that they can't receive labor analgesia. (Page 4, Line 59–Page 5, Line 60)

Results:

8. “When the population was restricted to only those who received LA, we found that pregnant women with the following four risk factors demonstrated a higher risk of IVD: advanced maternal age, lower maternal height, larger estimated fetal weight, and primipara (Table 3).” To be fair, you should do the same analysis on those who did NOT receive analgesia because you might find the same risk factors for operative delivery, so the analgesia may not have impacted this as much as you propose.

9. Indeed, for advanced maternal age and some other factors, analyzing only those with analgesia has a lower aOR than when you included everyone. Please also do the same analysis on those without analgesia.

Response to the Reviewer:

As suggested, we performed the same analysis as follows. In addition, some modification was required in the analysis data.

Among patients without labor analgesia, only primiparous had higher AOR, whereas, among those with labor analgesia, not only primiparous but also advanced maternal age are associated with increased risk of IVD. The results of the analysis are provided in this letter and some have been added to Table 3 in the revised manuscript.

10. The discussion mentions that operative delivery is associated with perineal tearing but this was not actually evaluated in this study. Please consider adding this analysis as it would help advise the results and discussion.

Response to the Reviewer:

Certainly, perineal tearing is one of the important complications of instrumental vaginal delivery. However, the outcome of the present study are the mode of delivery and the neonatal outcomes, and the complications that occur after delivery was not our interest. In fact that the perineal tearing is highly recognized as a complication of instrumental vaginal delivery is referred in the manuscript [20].

Discussion:

11. The aforementioned additional analysis on those who did NOT receive analgesia must be performed. These results should be reviewed after this sentence: “Among those who received LA, all four factors of advanced maternal age, lower maternal height, larger estimated fetal weight, and primipara increased the risk of IVD; the AOR of primipara was highest at 6.76 (95%CI 5.10–8.96) and that of advanced maternal age was second highest at 1.72 (95%CI 1.43–2.06).”

Response to the Reviewer:

Please refer the response to Comment 8 and 9 above.

12. I remain unconvinced of the conclusions but await additional analyses.

Response to the Reviewer:

We have made corrections as described above. We appreciate your re-review.

Reviewer #4:

In the Abstract

Adjusted Odds Ratio - put the confidence interval in the results. Introduction Last paragraph - just put the purpose of the study. It is not the place to describe the methodology.

Response to the Reviewer:

We followed the advice and revised. (Page 2, Line 21–23)

In Materials and Methods

Describe inclusion and exclusion criteria. Figure 1 contains the exclusion criteria and does not have the inclusion ones. Both should be described in the methodology.

Response to the Reviewer:

We followed the advice and revised. (Page 6, Line 66–77)

Lines 62 to 65 - the included text is disconnected from the rest.

Response to the Reviewer:

We confirmed that the submission guideline stated, "Figure captions must be inserted in the text of the manuscript, immediately following the paragraph in which the figure is first cited (read order).". We would appreciate if the reviewer could point out specifically if we seem to be mistaken.

Bibliographic references

Standardize the presentation of bibliographic references.

Reference 19 - has the year of publication in 2 locations.

As can be seen below: 19.Wong CA. (2014). Epidural and spinal analgesia/Anesthesia for labor and vaginal delivery, Obstetric Anesthesia: Principles and Practice. In: Chestnut DH (ed). Anesthesiology. Mosby: Maryland Heights, 2014, pp 490.

Response to the Reviewer:

We followed the advice and revised. (Page 25, Line 331–333)

Reviewer #5:

In general, the manuscript is fit for publication. In discussion section, data should be analyzed carefully, and conclusion should be made cautiously. Adverse neonatal and fetal outcome, cesarean section and instrumental vaginal delivery may be strongly associated with and even may be caused by advanced maternal age, lower maternal height, larger fetal weight, and primipara, and other risk factors, but not by LA. Perhaps, LA should not be restricted or prohibited in these pregnant women and those in labor with the four kinds of risk factors mentioned above. This should be re-analyzed carefully and added into the discussion section.

Response to the Reviewer:

We appreciate the reviewer’s advice. We did the same analysis on those without labor analgesia. The results of the analysis are provided in this letter and have been added to Table 3 in the revised manuscript.

As the reviewer’s advice, the aim is to counsel patients and allow them to make informed decision. We followed the advice and revised. (Page 2, Line 17–18, Page 4, Line 59–Page 5, Line 60)

Reviewer #6:

Page 5 line 63 and 64 : sentence are not clear. It is better to write the criteria inclusion in narrative.

Response to the Reviewer:

We followed the advice and revised.

Page 8 line 105 = table title not complete should be completed with place, and when?, n=??

Page 9 line = 110-111 should be deleted, should not be written here.

Page 9 line 125 : table title not complete should be completed with place, and when?, n=??

Page 10 line 127-128 should be deleted, should not be written here.

Page11 line 137-138 table title not complete should be completed with place, and when?, n=??

Page 11 line 140 and page 12 line 141 should be deleted, should not be written here

Response to the Reviewer:

We appreciate the reviewer’s peer review. We adhere to the guidelines when submitting. We humbly request your further advice.

Page 11 line 149 sentence is not clear

Response to the Reviewer:

We followed the advice and revised. (Page 14, Line 163–164)

Page 17 Line 217-222; sentence and statement is not clear

Response to the Reviewer:

We followed the advice and revised. (Page 19, Line 229–230)

Sincerely yours,

Shu Eguchi

Department of Pediatrics

St. Luke’s International Hospital
