# Supplementary material for: Impact of labor analgesia on mode of delivery and neonatal outcomes in Japan: A retrospective cohort study
Source: PLoS One. 2023 Apr 12;18(4):e0284368. doi: 10.1371/journal.pone.0284368 (PMC10096281; doi:10.1371/journal.pone.0284368)
Supplement: Attachment — Submitted filename: PONE-D-22-18968_R1 reviewed.docx [file pone.0284368.s005.docx]

**Reviewed:**

Page 2 (Abstract) line please add recommendation and Keywords

Page 3 line 57 – 60 : The statement is not appropriate in the introduction. this has to be stated in the methodology .

Author should write the research question and the research's goal at the end of introduction section.

Page 7 line 81-82 : not clear why the criteria incusion : age older than 35 year??

Line 90: Outcomes and definition should be Hypothesis?

Page 10 line	119	: Table title should be completed: what, where??how many ?

Line 122-125 (page 11) : no need in this abbreviation under table (it is already in the methodology)

Page 11 line 137: Table title should be completed: what, where??how many ? and should not separated with the table (in the same page).

Page 12 line 139-142: no need in this abbreviation under table (it is already in the methodology)

Page 12 line 146-147 : This statement should be in the methodology as hypothesis of this study.

Page 13 line 151-152 : Table title is not complete (what, when, where and how many)

Page 14 line 154-155: no need in this abbreviation under table (it is already in the methodology)

Page 20 line 249-250 : what you mean pregnant women with these risk factors??

Page 20 Line 252-256 : the statement is not clear

Page 21 line 274-276: the statement is not clear
