# Supplementary material for: Impact of labor analgesia on mode of delivery and neonatal outcomes in Japan: A retrospective cohort study
Source: PLoS One. 2023 Apr 12;18(4):e0284368. doi: 10.1371/journal.pone.0284368 (PMC10096281; doi:10.1371/journal.pone.0284368)
Supplement: Attachment — Submitted filename: Response to Reviewers.docx [file pone.0284368.s006.docx]

Dear Emily Chenette, Editor-in-Chief of *PLOS ONE*

On behalf of all co-authors of this manuscript, I would like to thank the editor and the reviewers for a thorough review of our manuscript and constructive advice, which have improved the manuscript considerably. We carefully went over the important comments/suggestions from the reviewers and revised the manuscript. Please find our point-by-point responses to the reviewers’ comments below. All changes we made in this revision are highlighted in yellow. We hope that you will find the revisions and comments satisfactory.

Reviewer #1:

#1. Approximately a quarter of all cases have needed some interventions such as IVD or ECS regardless of LA in this study. The number of ECS was fewer in LA group compared with non-LA group. In contrast, the Apgar score at 1 min was lower in LA group. You already discussed about the discrepancy, such as the use of fentanyl, however, further analysis should be necessary.

Response to the Reviewer:

We appreciate the reviewer’s peer review. Prior et al. reported that although the Apgar score was lower in infants delivered by IVD than in those delivered by CS, the reason is still unknown [22]. (Page 18, Line 240–241) Kumar et al. also reported that the effects of maternal epidural analgesia on neonatal respiration are not well described to date [11]. (Page 17, Line 215–218) In the present study, as well as other literatures, the discrepancy was unknown. (Page 17, 213–215)

As you mentioned, the indication of IVD or ECS was only determined by the fetal head station simply. I am confusing whether the fetal head station may influenced to the low Apgar score. Furthermore, we would like to know the reasons to decide the immediate delivery, for example, maternal factor or fetal factor. As you have abundant information over 5,000 valuable cases, more detail should be discussed as a final job in this study.

The indications for the immediate delivery related to maternal factors included gestational hypertension, eclampsia, placental abruption and HELLP syndrome. The indications related to fetal factors included fetal growth restriction and non-reassuring fetal status. The final decision was made by the obstetrician.

We followed the advice and add the above sentences. (Page 7, Line 106­–109)

In addition, you should correct the sentence in L73. We may not be able to understand the meaning, ‘cases in which LA was unavailable for LA’.

We followed the advice and revised. (Page 5, Line 75)

Reviewer #6:

Page 2 (Abstract) line please add recommendation and Keywords

Response to the Reviewer:

We appreciate the reviewer’s peer review. We followed the advice and add Keywords following the abstract. (Page 2, Line 27–28)

Page 3 line 57 – 60 : The statement is not appropriate in the introduction. this has to be stated in the methodology .

Author should write the research question and the research's goal at the end of introduction section.

We followed the advice and revised. (Page 4, Line 58–62)

Page 7 line 81-82 : not clear why the criteria inclusion : age older than 35 year??

The inclusion criteria did not contain maternal age. The data obtained included maternal age to dichotomize as advanced maternal age.

Line 90: Outcomes and definition should be Hypothesis?

We followed the advice and revised. (Page 6, Line 91)

Page 10 line 119 : Table title should be completed: what, where??how many ?

We followed the advice and revised. (Page 9, Line 125–126)

Table 1. Patients characteristics and association between Labor analgesia group and Non-labor analgesia group at obstetrics inpatients. (N=5184)

Line 122-125 (page 11) : no need in this abbreviation under table (it is already in the methodology)

We followed the advice and revised. The abbreviations were removed, but analysis methods were left as they are not described in the methodology.

Page 11 line 137: Table title should be completed: what, where??how many ? and should not separated with the table (in the same page).

We followed the advice and revised. (Page 11, Line 141)

Table 2. Association among mode of delivery at obstetrics inpatients. (N=5184)

Page 12 line 139-142: no need in this abbreviation under table (it is already in the methodology)

We followed the advice and revised. The abbreviations were removed, but analysis methods were left as they are not described in the methodology.

Page 12 line 146-147 : This statement should be in the methodology as hypothesis of this study.

We followed the advice and revised. (Page 6, Line 92–94)

Page 13 line 151-152 : Table title is not complete (what, when, where and how many)

We followed the advice and revised.

Table 3. The risk of labor analgesia against instrumental vaginal delivery and emergency cesarean section, and adverse neonatal outcomes at obstetrics inpatients. (N=5184)

Page 14 line 154-155: no need in this abbreviation under table (it is already in the methodology)

We followed the advice and revised. (Page 12, Line 150–151)

Page 20 line 249-250 : what you mean pregnant women with these risk factors??

We followed the advice and revised. (Page 19, Line 247)

Page 20 Line 252-256 : the statement is not clear

We followed the advice and revised. (Page 19, Line 249–250)

Page 21 line 274-276: the statement is not clear

We followed the advice and revised. (Page 20, Line 272–273)

In addition, it is difficult to establish the cut-off for maternal height and estimated birth weight and decide clinical practice depend on the cut-off.

Sincerely yours,

Shu Eguchi

Department of Pediatrics

St. Luke’s International Hospital
