# Supplementary material for: Impact of labor analgesia on mode of delivery and neonatal outcomes in Japan: A retrospective cohort study
Source: PLoS One. 2023 Apr 12;18(4):e0284368. doi: 10.1371/journal.pone.0284368 (PMC10096281; doi:10.1371/journal.pone.0284368)
Supplement: Attachment — Submitted filename: Response to Reviewers.docx [file pone.0284368.s007.docx]

Dear Emily Chenette, Editor-in-Chief of *PLOS ONE*

On behalf of all co-authors of this manuscript, I would like to thank the editor and the reviewers for a thorough review of our manuscript and constructive advice, which have improved the manuscript considerably. We carefully went over the important comments/suggestions from the reviewers and revised the manuscript. Please find our point-by-point responses to the reviewers’ comments below. All changes we made in this revision are highlighted in yellow. We hope that you will find the revisions and comments satisfactory.

1) Short title: Impact of labor analgesia on delivery and neonates. "I suggest: Impact of labor analgesia on delivery"
Response to the Reviewer:

We appreciate the reviewer’s peer review. We followed the advice and revised. (Page 1, Line 3)

2) In the discussion section there are too many "our study, our results". It makes the reading a little bit tiresome. I would suggest use "In the present study..." for example.

We followed the advice and revised.

3) I strongly suggest a full revision by a english native speaker.

We followed the advice. The latest manuscript was revised by English native speakers.

Sincerely yours,

Shu Eguchi

Department of Pediatrics

St. Luke’s International Hospital
